# Exploring the potential cost-effectiveness of a new computerised decision support tool for identifying fetal compromise during monitored term labours: an early health economic model

**DOI:** 10.1186/s12962-024-00580-x

**Published:** 2024-10-07

**Authors:** H. E. Campbell, S. Ratushnyak, A. Georgieva, L. Impey, O. Rivero-Arias

**Affiliations:** 1 National Perinatal Epidemiology Unit, Nuffield Department of Population Health, University of Oxford, Oxford, UK; 2 Oxford Labour Monitoring Group, Nuffield Department of Women’s and Reproductive Health, University of Oxford, Oxford, UK; 3 Fetal Medicine Unit, John Radcliffe Hospital, Oxford, UK

**Keywords:** Fetal monitoring, Decision support tool, Fetal compromise, Early economic model, Economic evaluation

## Abstract

**Background:**

Around 60% of term labours in the UK are continuously monitored using cardiotocography (CTG) to guide clinical labour management. Interpreting the CTG trace is challenging, leading to some babies suffering adverse outcomes and others unnecessary expedited deliveries. A new data driven computerised tool combining multiple clinical risk factors with CTG data (attentive CTG) was developed to help identify term babies at risk of severe compromise during labour. This paper presents an early health economic model exploring its potential cost-effectiveness.

**Methods:**

The model compared attentive CTG and usual care with usual care alone and simulated clinical events, healthcare costs, and infant quality-adjusted life years over 18 years. It was populated using data from a cohort of term pregnancies, the literature, and administrative datasets. Attentive CTG effectiveness was projected through improved monitoring sensitivity/specificity and potential reductions in numbers of severely compromised infants. Scenario analyses explored the impact of including litigation costs.

**Results:**

Nationally, attentive CTG could potentially avoid 10,000 unnecessary alerts in labour and 2400 emergency C-section deliveries through improved specificity. A reduction of 21 intrapartum stillbirths amongst severely compromised infants was also predicted with improved sensitivity. Attentive CTG could potentially lead to cost savings and health gains with a probability of being cost-effective at £25,000 per QALY ranging from 70 to 95%. Potential exists for further cost savings if litigation costs are included.

**Conclusions:**

Attentive CTG could offer a cost-effective use of healthcare resources. Prospective patient-level studies are needed to formally evaluate its effectiveness and economic impact in routine clinical practice.

**Supplementary Information:**

The online version contains supplementary material available at 10.1186/s12962-024-00580-x.

## Background

 In the UK, around 60% of labours are continuously monitored with cardiotocography (CTG), which graphically displays uterine contractions and the fetal heart rate [1–3]. Clinicians perform visual assessments of the CTG trace to identify signs of fetal oxygen deprivation and if the baby appears to be at risk of compromise, they can expedite the birth by Caesarean section or instrumentally. However, difficulty in interpreting CTG patterns results in intra and inter observer variability and because the physiology of fetal oxygenation in complicated labours with multiple risk factors is poorly understood, this means that the full potential of CTG for improving neonatal outcomes has yet to be reached [1, 4]. In the UK, around 1200 healthy term babies suffer avoidable morbidity (asphyxia and brain damage) and mortality each year, whilst many more are born unnecessarily by emergency Caesarean Sects. [5, 6]. These implications have profound consequences; in 2018–19, the National Health Service (NHS) paid around £1.2bn in maternity-related negligence claims (nearly 1% of the entire NHS budget) with most claims related to shortcomings in fetal monitoring and labour management (overall such claims are small in number but high in value) [7, 8]. Further, an estimated 3,000 quality-adjusted life years (QALYs) are lost annually to cerebral palsy caused by avoidable oxygen deprivation [9]. The need for improvements in the safety and quality of maternity care is reflected by various UK initiatives including NHS England’s ‘Saving Babies’ Lives’ programme and the ‘Each Baby Counts’ programme overseen by the Royal College of Obstetricians and Gynaecologists (RCOG), both of which aimed to reduce adverse outcomes for babies during labour and childbirth [5, 10, 11].

In recent years a number of computerised decision support systems have been developed which aim to act as an interface by collating, presenting and interpreting relevant information relating to the CTG and some routinely collected clinical data (e.g. partographs, maternal vital signs, maternal anaesthesia and analgesia) [1, 12, 13]. Such systems make an assessment of these data and compare overall CTG patterns observed against established guidelines or clinician defined criteria on CTG interpretation. Patterns indicative of oxygen deprivation/compromise lead to an alert being raised. Clinical trials have shown a number of these systems do not improve neonatal or longer-term infant outcomes and are unlikely to represent a cost-effective use of scarce healthcare resources [1, 2, 12, 14]. Reasons for the lack of effect likely include system reliance upon clinician expertise/established guidelines for interpreting CTG patterns, both of which have the limitations documented above [2, 15, 16].

Given that the benefit of clinical care CTG interpretation is poorly proven, scientists have been developing a new computerised decision support tool (referred to henceforth as attentive CTG). Attentive CTG differs from previous systems in that it utilises Machine Learning techniques and/or prognostic models developed from large, administrative patient-level datasets, to facilitate individualised predictions of the risk of fetal compromise (a composite of stillbirth, neonatal death, neonatal encephalopathy, seizures, and resuscitation followed by admission to neonatal intensive care for > 48 h) [17, 18]. From an initial concept developed using data from 7500 births (version 1.0), attentive CTG has evolved over time, with subsequent versions developed using larger patient databases and incorporating multiple clinical risk factors relating to the mother, the pregnancy, and the labour, alongside the CTG data. Different versions of the attentive CTG tool have been externally validated on datasets both within and outside of the UK and have been shown to identify more compromised babies than current practice alone [19–24]. The tool also appears to facilitate a reduction in unnecessary labour alerts and costly and invasive emergency Caesarean section deliveries. The newest version (3.0) will re-estimate prognostic models using data on over 100,000 term births monitored at the John Radcliffe Hospital in Oxford between 1993 and 2022.

Based upon the work conducted thus far, attentive CTG would appear to offer a means of improving infant outcomes at the time of labour and birth. However, a formal demonstration of the effectiveness (and cost-effectiveness) of the system by an adequately powered prospective patient-level study would be a necessary pre-requisite to any recommendations being made about the use of the system within routine NHS maternity care. As such studies can be hugely costly and time consuming to conduct, prior to making an investment in such research, it is pertinent to consider what the potential costs and health consequences of its use might be if the predicted improvements in prognostic accuracy could be realised in routine practice. If effectiveness and value for money is unlikely, then progression to prospective evaluation is unnecessary. Early health economic modelling has been utilised for this purpose in a number of disease areas [25, 26]. By modelling the costs and outcomes of a routine care pathway, and how an intervention could potentially modify this pathway, such a model can provide valuable information on potential cost-effectiveness and can inform next steps in the evaluation process. This paper reports on the development and findings of such an early health economic model exploring the potential cost-effectiveness of attentive CTG for identifying fetal compromise during term labours.

## Methods

We developed and populated an early health economic model to simulate and compare the potential costs and health outcomes of attentive CTG added to usual labour care, and usual labour care alone, for term women (at least 37 + 0 weeks of gestation completed) undergoing CTG monitoring during labour.

### Model structure

**Fig. 1:**
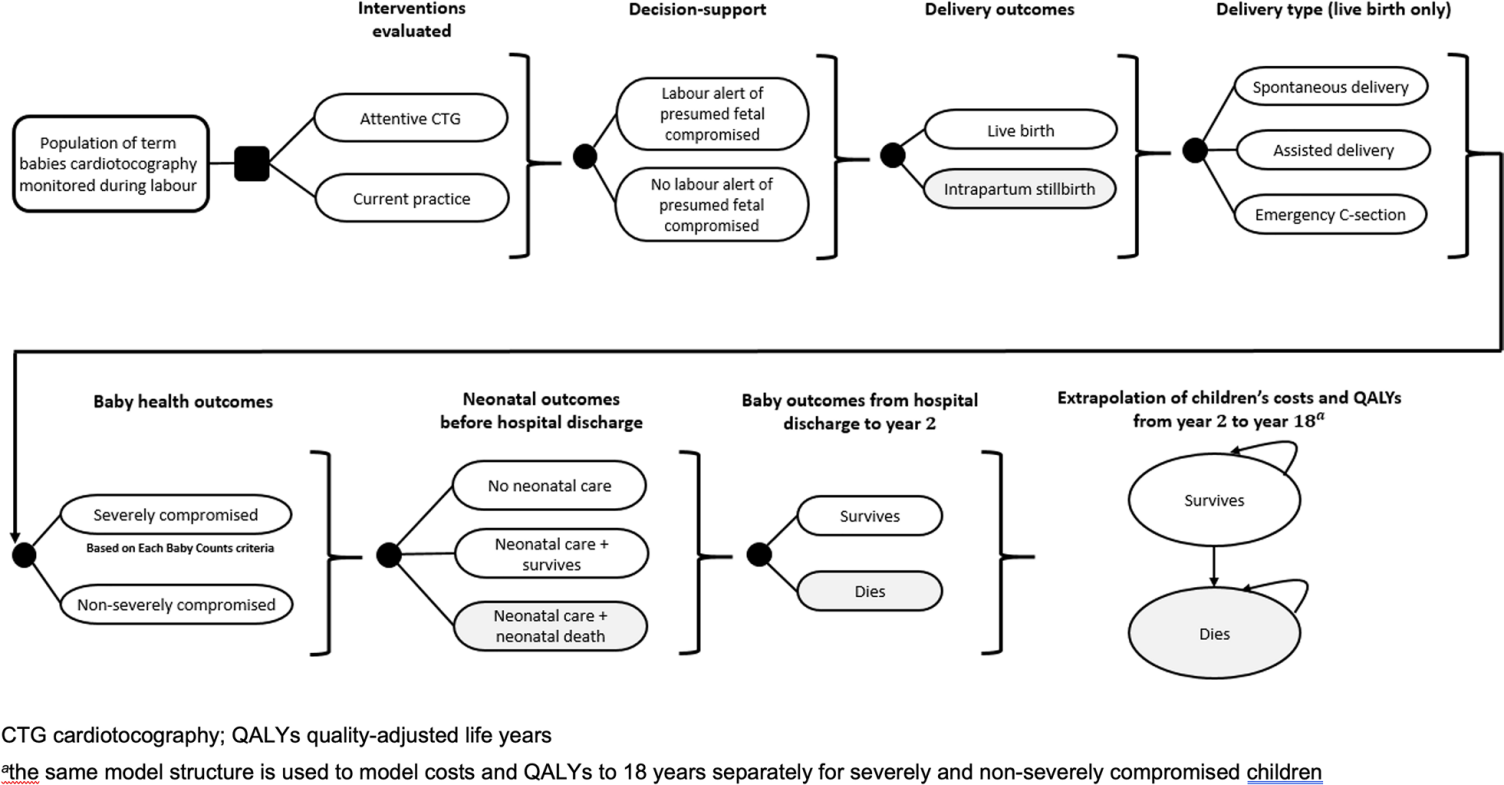
Structure of the early health economic model

The model was hybrid in nature comprising decision tree and Markov components and was developed through collaborative work between the project’s clinical and health economic teams. A decision tree uses a series of branches to map out events along a clinical pathway and is well suited to modelling non-repetitive events over short time periods. Figure 1 illustrates the model and shows the key events arising between labour monitoring and two years post-birth. Within the tree, individuals move along pathways chronologically according to whether there has been an alert for presumed fetal compromise during labour, the subsequent birth outcome (live birth or stillbirth), and for live births, the mode of birth (spontaneous, assisted, or emergency caesarean section). The infant’s live birth outcome (severely or not severely compromised) is then modelled, followed by the need for neonatal unit care, and the infant’s survival status at hospital discharge and two years post-birth. The definition of severe compromise for the economic model was based upon that devised for the ‘Each Baby Counts’ programme to reduce the number of babies who die or are left severely disabled as a result of incidents occurring during term labour [5]. Supplementary Table S1 lists these criteria, which were also used to define severe compromise as predicted by the attentive CTG tool.

Beyond two years, simple Markov models were used to simulate the longer-term costs and outcomes of surviving infants who had and who had not suffered severe compromise. As shown in Fig. 1, these models comprised just two health states (survives and dies) and each year infants in the ‘survives’ health state face a probability of death (dependent upon their compromise status).

The model structure shown in Fig. 1 was used for both the attentive CTG and usual care arms, with the potential impact of attentive CTG being explored by altering various pathway probabilities as described in a following sub-section. The cohort entering the model were individuals undergoing continuous fetal heart rate monitoring during a term labour. UK data suggest that this is approximately 60% of labours, which in England during 2021, would have equated to around 329,361 of the 548,935 term deliveries recorded [27, 28]. The model was initially constructed and run in TreeAge [29] and subsequently replicated in Microsoft Excel [30].

The analysis took the forms of cost-consequence and cost-utility analyses from the perspective of the NHS in England and utilised a time horizon that followed infants up to 18 years of age. Health care resource use associated with monitoring, birth and subsequent infant care were included, with NHS litigation costs estimated for a secondary analysis. Unit costs to value resource use were taken from national databases and the published literature and were expressed in 2020/21 UK pounds [31]. Various health outcomes were reported including the numbers of labour alerts for presumed fetal compromise, emergency caesarean section deliveries, intrapartum stillbirths, neonatal deaths, and severely compromised infants and all infants alive at 2 years. Infant quality adjusted life years (QALYs) which allow the life years lived by an individual to be adjusted for the levels of quality of life they experience, were also modelled for each monitoring approach. Costs and QALYs arising after the first year in the model were discounted at the recommended rate of 3.5% [32].

### Model inputs

Model inputs are described briefly below, with full detail provided in the supplementary file under analogous headings.

#### Event probabilities

Conditional event probabilities for the decision tree were estimated predominantly from electronic patient record (EPR) data on outcomes observed during and following 22,833 monitored term labours at the John Radcliffe Hospital in Oxford between 2013 and 2018. Supplementary Table S2 shows these probabilities which were used to populate the tree up to infant hospital discharge. Baby survival between hospital discharge and two years was conditional upon health outcome at birth (severely or not severely compromised) and was informed by national statistics and the published literature [33, 34].

Amongst the severely compromised babies alive at two years and entering the ‘survives’ health state of the Markov model, a proportion would have been left with neurodevelopmental disabilities. We used data from the TOBY trial evaluating therapeutic cooling for babies suffering perinatal asphyxia at birth to estimate this proportion, annually, up to the age of 18 [33, 35]. Infants with neurodevelopmental disabilities were then assumed to face age adjusted annual mortality risks equivalent to those observed for normal birthweight infants with cerebral palsy [36]. Mortality for those without disability and those not severely compromised was modelled using age adjusted life table data for England [37, 38]. See the supplementary file and columns 2 and 3 of supplementary Table S3.

#### Health-related quality of life

The published literature informed annual quality of life weights within the Markov model for surviving infants who had and who had not been severely compromised at birth (see supplementary file and columns 4 and 5 of supplementary Table S3) [39, 40]. Few data are available on the health-related quality of life of infants below the age of 2 years and so we assumed levels of quality of life during the first two years of the model for severely / non-severely compromised babies, would be at the same levels as estimated at year two.

### Unit costs

Included in the decision tree were the costs of the various delivery types (spontaneous, assisted, or emergency C-section) and of neonatal unit (NNU) admissions. These costs were estimated to be conditional upon the outcome of the baby as described in the supplementary file and shown in Table S4.

For infants surviving to 2 years, costs from hospital discharge were estimated according to whether an infant had been severely compromised and/or had required NNU care. Costs were included for routine post-hospital discharge community care (baby review and development contacts) and for expected primary and secondary healthcare consumption during the first two years of life (see supplementary file and Table S4) [41–43]. Costs for severely compromised infants who died between hospital discharge and Year 2, were informed by the TOBY trial [44], and for the small number of non-compromised babies who died before two years, using a survival-based costing approach (see supplementary file and Table S4) [31, 34, 41–43, 45]. Emergency ambulance transfer and post-mortem costs were included for both groups [31, 46].

For surviving infants entering the Markov models at the end of year 2, the published literature was used to estimate annual costs up to 18 years of age for those who had/had not been severely compromised at birth (see supplementary file and columns 6 and 7 of Table S3) [44, 47] .

### Modelling the addition of attentive CTG to current practice

**Fig. 2:**
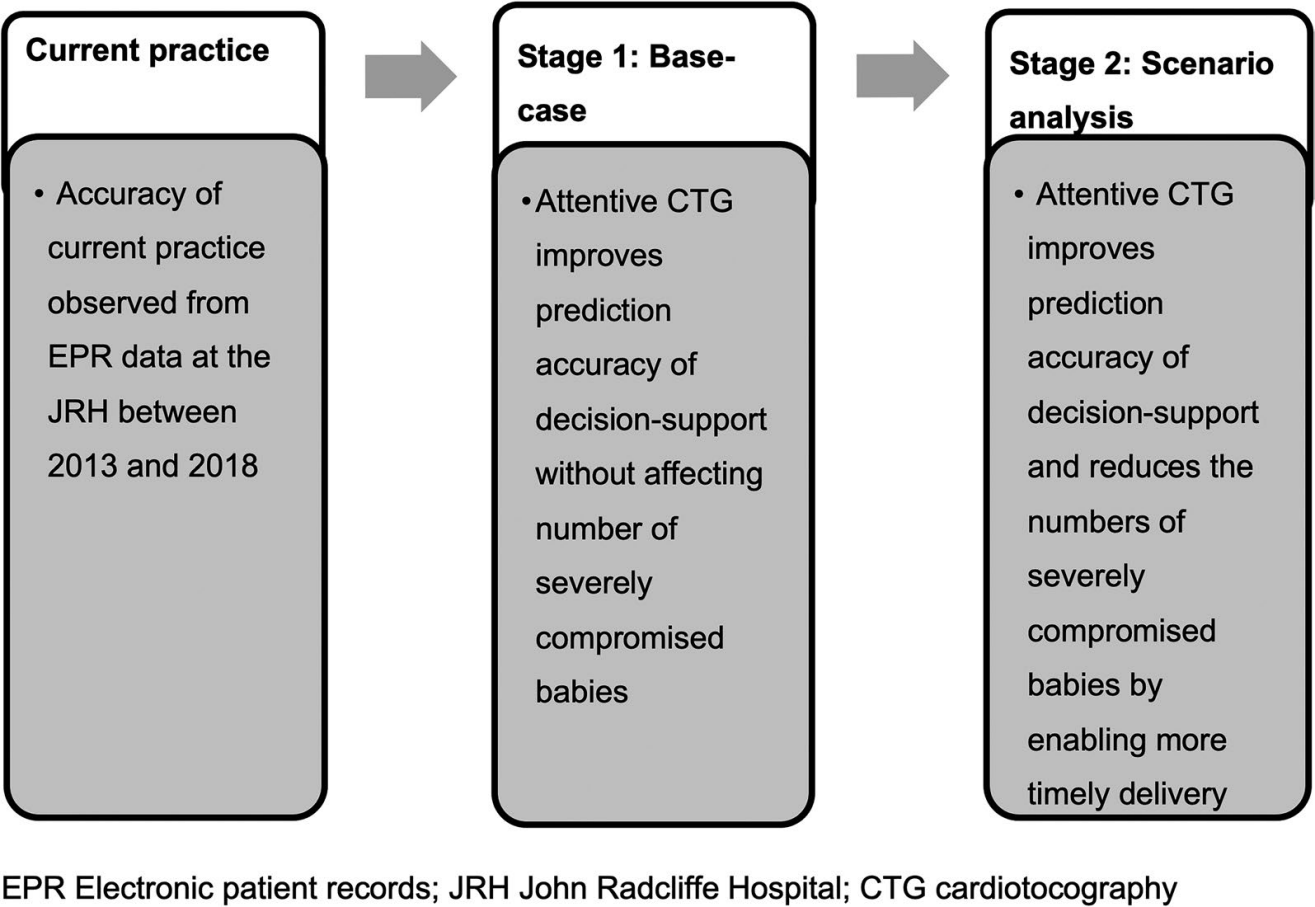
Model workflow and stages describing assumptions made during the evaluation of attentive CTG

A two-stage process was used to model the potential impact of attentive CTG (see Fig. 2). In stage 1 (referred to as the base-case analysis) the ability of the aid to more accurately predict which babies would and would not suffer severe compromise (but not to alter this outcome) was modelled. Estimates of improved prediction accuracy (preliminary results for version 3.0 of the attentive CTG tool were provided by the clinical team and were based on data simulations with a retrospective dataset of over 60,000 term births) were used to model how labour alerts within the 2013–2018 John Radcliffe Hospital dataset would have been altered if attentive CTG had been added to routine practice.

By way of illustration, the first row of Table 1 presents the observed combinations of alert status (alert / no alert) and compromised status (baby severely compromised/not compromised) for the 22,833 monitored labours in the dataset. The sensitivity of current practice was estimated at 0.38, with 38 of the 101 severely compromised infants in the dataset having an alert correctly raised during labour, i.e. having been recorded as undergoing an operative birth for the primary documented reason of presumed fetal compromise (true positive). Specificity was 0.87, with no alert for 19,748 of the 22,732 infants not severely compromised (true negative). The third row of Table 1 shows the predicted re-classification of the cohort, with anticipated improvements in sensitivity and specificity with attentive CTG to 0.55 and 0.90 respectively. For the stage 1 modelling, we assumed being able to detect more of the severely compromised babies with the tool would not ultimately alter the compromised status of these babies and so movement would only be from column B to A in Table 1. The model did however assume that the birth outcome (live birth or stillbirth), type of birth (spontaneous, assisted, or emergency C-section), and neonatal mortality for these re-classified women would change to match those of other women in the dataset who had received a true positive alert. The supplementary file (Tables S5 and S6) provide detail of how birth outcomes and types were altered within the model for re-classified women. Changes to birth outcome and type were also assumed for women without a severely compromised baby who moved from an unnecessary alert to a no alert status (moving from column C to column D in Table 1) as a result of the improved specificity with attentive CTG. The base-case analysis can be considered conservative in nature, with improvements in prediction accuracy and changes to birth management not leading to reductions in the risk of severe compromise.

**Table 1:** Current practice labour alert and baby compromised classifications and numbers of alerts^a, b^

	(Alert & SC) (A)	(No Alert & SC) (B)	Total SC (A + B)	Sensitivity (A/(A + B))	(Alert & NSC) (C)	(No Alert & NSC) (D)	Total NSC (C + D)	Specificity (D/(C + D))	Total Alerts (A + C)
Current practice	38	63	101	**0.38**	2,984	19,748	22,732	**0.87**	3,022
Stage 1 analysis ^c^									
Base-case	56	45	101	**0.55**	2,273	20,459	22,732	**0.90**	2,329
Stage 2 analysis ^d^									
25% reduction	52	45	97		2,278	20,459	22,737		2,329
50% reduction	47	45	92		2,282	20,459	22,741		2,329
75% reduction	43	45	88		2,287	20,459	22,746		2,329
100% reduction	38	45	83		2,291	20,459	22,750		2,329

^a^For the John Radcliffe Hospital cohort

^b^Also shown are the implications of potential improvements in alert sensitivity and specificity with attentive CTG (Stage 1), and with subsequent reductions in the risk of severe compromise for additionally identified babies (Stage 2)

^c^The improved sensitivity with attentive CTG enables the identification of more compromised babies during labour, thus leading to altered delivery types and delivery outcomes (see Fig. 1 and supplementary Tables S5 and S6) but does not alter the overall number of severely compromised babies. Movement is between column B ‘current practice’ to column A ‘base-case’

^d^The improved sensitivity with attentive CTG and altered delivery management does lead to reductions in the risk of severe compromise for the additionally identified babies. Risk reductions of varying magnitudes are modelled. Movement is between column B ’current practice’ to column A ‘% reduction’’ and column C ‘% reduction’

*SC* severely compromised, *NSC* non-severely compromised

With Stage 2, we extended the modelling further and simulated that the predicted changes to the delivery management of women with affected babies brought about by the improved sensitivity with attentive CTG, would have reduced the risk of severe compromise at birth. In the absence of data to inform the magnitude of this risk reduction, we modelled a range of scenarios with risk reductions of 25%, 50%, 75% and 100%.

Aided by the project team, we considered the various cost components likely to be associated with attentive CTG (purchase of the application, initial system set-up and training, and subsequent ongoing annual IT support and training). Preliminary estimates of these costs for the 130 NHS Trusts in England that would potentially use the tool were estimated and then divided through by the total number of monitored labours in England per year (329,361). For the initial set-up costs we divided through by the expected number of monitored labours in England over five years, acknowledging that this initial investment cost should be apportioned across more labours than just those during a given year. The resulting cost of attentive CTG per monitored labour used within the model was £13.22.

### Statistical analysis

Where data permitted, event probabilities, costs and quality of life weights were entered into the model as distributions rather than as point estimates, so as to facilitate probabilistic sensitivity analysis (PSA) [48]. PSA enables the analyst to assess the impact on the model’s results of the joint uncertainty across the model inputs, and is implemented by running the model a large number of times (in this case 10,000 times), each time randomly sampling a set of parameter values from the distributions and re-calculating the results. This generated a distribution of possible clinical outcomes, and cost and effect estimates for attentive CTG and for current practice alone. To assess the validity of the model outputs, we compared numbers of key clinical outcomes from the current practice arm of the model with those reported at the national level by a range of established sources [5, 28, 49].

For each year of the model, discounted costs and QALYs were modelled, before being summed to generate an estimate of total costs and QALYs. Mean (standard error) discounted total healthcare costs and QALYs to 18 years of age for each arm of the model were then compared using mean differences and 95% parametric confidence intervals around the differences. Cost-effectiveness was expressed using the incremental net monetary benefit, which converts the additional health gain from an intervention (here QALYs) into a monetary value using an established amount considered to represent society’s maximum willingness to pay for a QALY (£25,000) and subtracts from this value the additional costs of using the intervention. If the resulting figure is positive, then the value of the health gains brought about by the tool would be considered greater than the incremental costs and its use would be considered cost-effective [50].

Uncertainty around the cost-effectiveness results was depicted using cost-effectiveness acceptability curves (CEACs) [51]. For a range of different values of maximum willingness to pay for a QALY, CEACs plot the percentage of the model’s 10,000 simulated PSA results that suggest attentive CTG is cost-effective (i.e. where the predicted incremental costs and QALYs generate a positive incremental net monetary benefit value).

### Scenario analyses

In addition to the base-case and stage 2 analyses described above, we performed a number of additional scenario analyses (see supplementary file for full details). Amongst these was a scenario in which litigation costs were included. For this we utilised data from the published literature and NHS Resolution to estimate that compensation of around £10 million would be paid by the NHS for a brain injury/case of cerebral palsy where clinical negligence was established. Also, that an estimated 10% of all parents of babies born with a brain injury make a claim to NHS Resolution, and that around 27% of these cases are likely to be successful (see supplementary file for further detail) [52, 53]. These data were used to model the likely impact upon cost-effectiveness if attentive CTG, could, through its improved sensitivity, alter delivery management, improve delivery and neonatal outcomes and reduce litigation costs.

## Results

### Base-case analysis

The first three rows of Table 1 show the predicted impact of including attentive CTG alongside usual labour care for the John Radcliffe Hospital cohort. Due to the small number of severely compromised infants (*n* = 101), an improvement in alert sensitivity from 0.38 to 0.55, was predicted to identify an additional 18 severely compromised babies (movement from ‘current practice’ column B to ‘base-case’ column A). A greater impact would come from the smaller improvement in specificity (0.87 to 0.9), with attentive CTG preventing an estimated 711 unnecessary alerts (moving women from ‘current practice’ column C to ‘base-case’ column D). The overall improvement in prognostic accuracy with attentive CTG was a predicted reduction of 693 labour alerts (final column of Table 1).

Table 2 illustrates the potential impact of the aid on key clinical events by scaling model predictions up to a national level for England. With the improvements to specificity, a reduction of 10,000 (22.94%) unnecessary labour alerts amongst women whose babies are not compromised, is predicted. This in turn could lead to almost 2,400 (4.26%) fewer C-section deliveries as the delivery management of these women is now less invasive (supplementary Table S6).

**Table 2:** Predicted clinical outcomes with attentive CTG and current practice, and current practice alone, for England^a^

Clinical outcomes	Current practice *N* (SE)	Attentive CTG *N* (SE)	Mean difference (95% confidence interval)	% change
Alerts for presumed fetal compromise	43,591 (736)	33,589 (648)	− 10,002 (-11,931 to -8,072)	− 22.94%
Emergency C-sections	56,273 (821)	53,878 (810)	− 2,396 (-4,663 to -128)	− 4.26%
Intrapartum stillbirths	72 (32)	51 (27)	− 21 (− 103 to 62)	− 28.70%
Neonatal deaths	303 (66)	304 (66)	+ 1 (− 181 to 183)	+ 0.36%
Severely compromised babies alive at 2 years	1,058 (123)	1,076 (125)	+ 18 (− 321 to 357)	+ 1.71%
All babies alive at 2 years	328,768 (76)	328,787 (74)	+ 19 (− 181 to 219)	+ 0.01%

^a^Base-case analysis for an annual cohort of 329,361 monitored term births in England – assuming 60% of the 548,935 annual deliveries in England in 2021 were monitored [28]

*SE* standard error, *CTG* cardiotocography, *C-section* Caesarean section

Table 2 also suggests that improvements in sensitivity with attentive CTG, could result in a predicted 28.70% reduction in intrapartum stillbirths, on account of an increased likelihood of having an expedited delivery following an alert now being correctly raised (supplementary Tables S5 and S6). In the base-case analysis, a direct consequence of these babies now being live born but still severely compromised is a small increase in neonatal deaths within the attentive CTG group, as more infants survive within the model to face this in-hospital mortality risk. Over a two-year period, there is a predicted 0.01% rise in overall surviving infants and a more noticeable 1.7% increase in surviving infants who were severely compromised at birth.

Supplementary Table S7 compares key events predicted by the model’s comparator arm with those reported at a national level by established sources. The model was able to predict live births and intrapartum stillbirths in line with those reported by the Each Baby Counts initiative. A direct comparison of the proportions of severely compromised infants predicted by the model and reported by Each Baby Counts however was not possible on account of the denominator for the Each Baby Counts estimate being all term births rather than monitored term births. Assuming that 60% of all births would have been monitored, then the prevalence of severe compromise would have been almost 0.3% in the Each Baby Counts cohort, and not wholly dissimilar to the 0.4% predicted by the model.

The top half of Table 3 shows the 18-year cost-effectiveness results for the base-case analysis. When compared with usual care alone, the addition of attentive CTG was associated with a non-significant per baby reduction in cost (−£46, 95% CI-£109 to £17) and a non-significant increase in QALYs (0.00049, 95% CI − 0.00791 to 0.00889). The cost reduction was driven by fewer unnecessary labour alerts resulting in reductions in assisted and C-section deliveries. The mean QALY gain was attributable to a small increase in the number of severely compromised babies surviving as a result of more accurate alerts and the expediting of deliveries, preventing intrapartum stillbirth.

**Table 3:** Mean (SE) per baby costs, QALYs and cost-effectiveness of attentive CTG compared with current practice^a^

Comparators	Mean (SE) cost discounted	Mean cost difference (95% confidence interval)	Mean (SE) QALYs discounted	Mean QALY difference (95% confidence interval)	Net monetary benefit (SE)
Base-case analysis					
Current practice	£16,697 (£327)	---	12.00139 (0.00311)	---	£283,338 (£337)
Attentive CTG	£16,651 (£328)	− £46 (− £109 to £17)	12.00188 (0.00308)	0.00049 (− 0.00791 to 0.00889)	£283,396 (£337)
Incremental net monetary benefit (95% confidence interval)					**£58 (− £171 to £288)**
Base-case analysis including litigation costs					
Current practice	£17,137 (£332)	---	12.00139 (0.00311)	---	£282,898 (£347)
Attentive CTG	£17,099 (£333)	− £38 (− £214 to £137)	12.00188 (0.00308)	0.00049 (− 0.00791 to 0.00889)	£282,948 (£348)
Incremental net monetary benefit (95% confidence interval)					**£51 (− £279 to £380)**

^a^Costs and QALYs are up to 18 years of age with results shown for the base-case analysis and a scenario analysis including litigation costs

*SE* standard error, *CTG* cardiotocography

Calculation of the INMB for the base-case analysis using the simulated mean cost and QALY differences (shown in supplementary Figure S1), suggested that the value of the health benefits gained from using attentive CTG outweighed the associated costs (Table 3). Uncertainty surrounded the INMB figure of £58 however, and estimation of the associated CEAC suggested that at a maximum willingness to pay of £25,000 per QALY, the probability of the tool representing a cost-effective use of healthcare resources was around 70%.

### Stage 2

The final four rows of Table 1 illustrate the potential implications for the John Radcliffe Hospital cohort, if attentive CTG *could* lead to a reduction in the risk of having a severely compromised baby amongst the additional women correctly identified as ‘at risk’. As the risk reduction is increased, more women move from ‘base-case’ column A to ‘stage 2’ column C in Table 1. A 100% reduction in risk would see all 18 additionally identified women move in this direction. Supplementary Tables S8 and S9 respectively show the changes to key clinical events, and the mean cost and QALY differences and INMBs, associated with each of these analyses. Cost savings, QALY gains, and the cost-effectiveness of attentive CTG increase with the prevention of more cases of severe compromise.

**Fig. 3:**
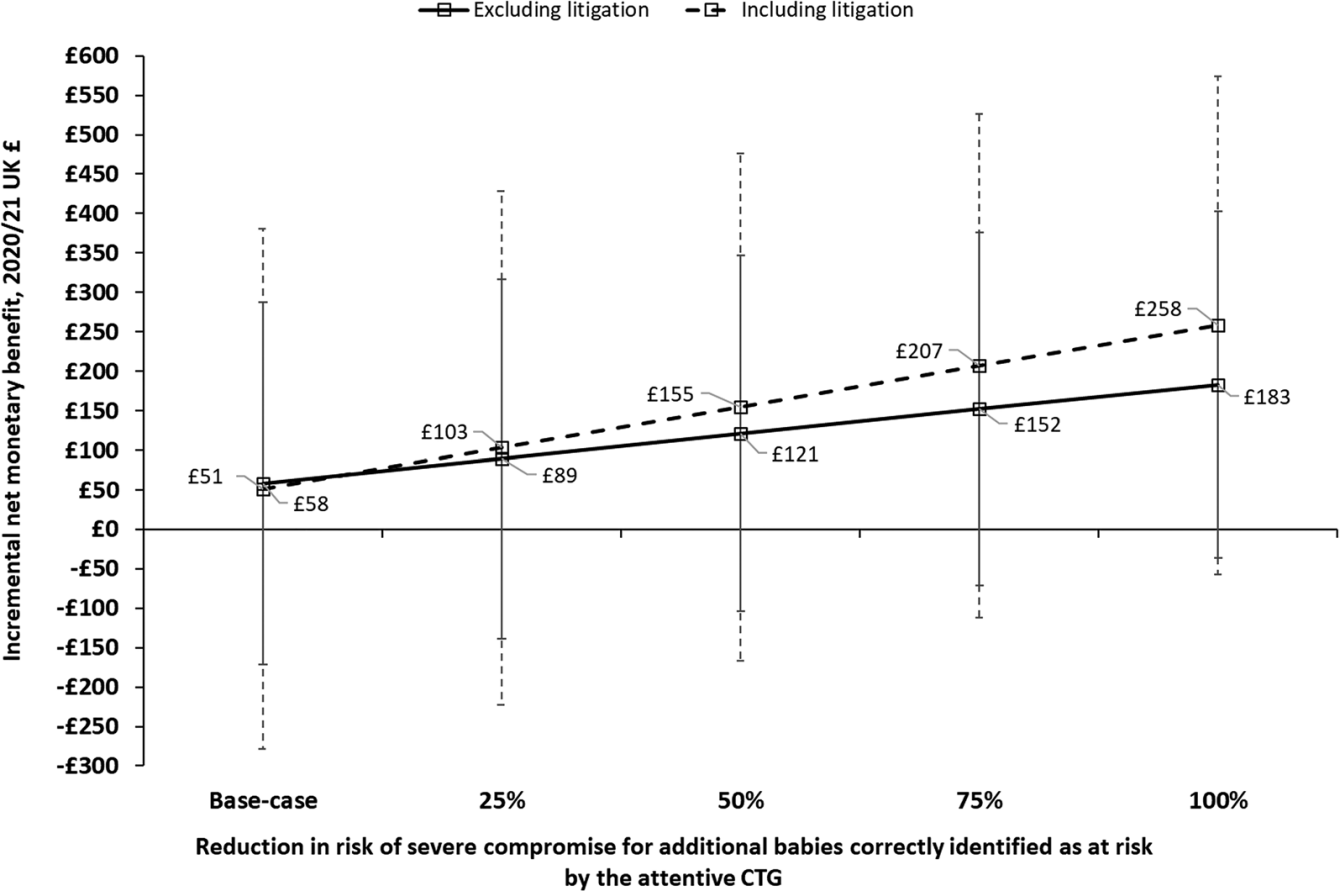
Incremental net monetary benefit and associated 95% confidence intervals with attentive CTG ^a^ ^a^Results shown for the model’s base-case analysis (with and without litigation costs) and the various stage 2 analyses

**Fig. 4:**
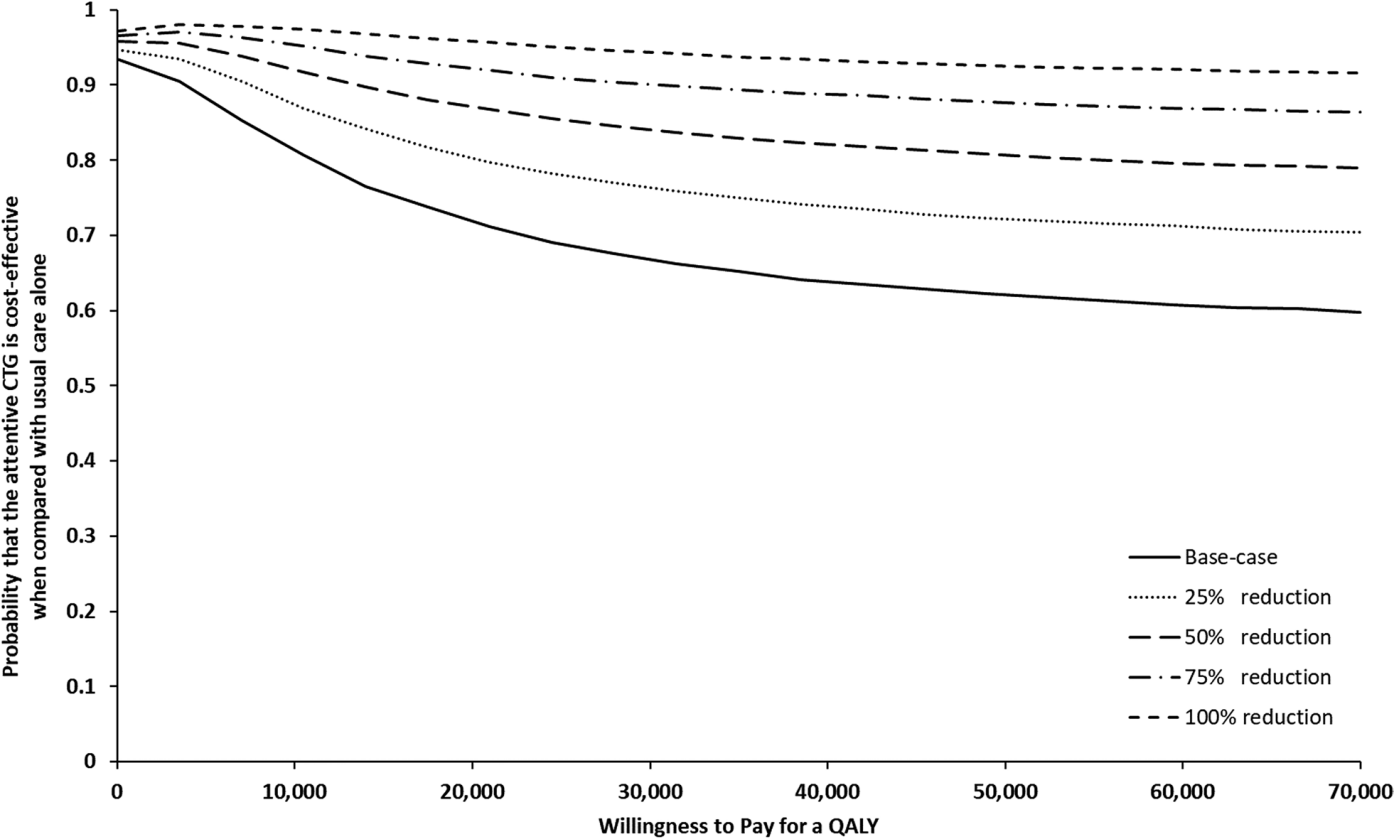
Cost-effectiveness acceptability curves plotting the likelihood that attentive CTG is cost-effective^a^ ^a^Curves shown are for the model’s base-case analysis and for the various stage 2 analyses exploring reductions in the risk of severe compromise for additional babies correctly identified as at risk

Figure 3 (solid lines) plots the INMB for the base-case analysis and each of the four stage 2 scenarios. Assuming the birth of a severely compromised baby could be prevented for 25%, 50%, 75%, and 100% of the additional women who would now receive an accurate alert with attentive CTG, increases the INMB to £89, £121, £152, and £183 respectively. The associated probabilities of cost-effectiveness shown by the CEACs in Fig. 4 are 78%, 85%, 91% and 95%.

### Scenario analysis results

The lower half of Table 3 shows the base-case cost-effectiveness results with litigation costs included. The mean cost saving per infant is reduced from − £46 to − £38 and with no change to health outcomes, the INMB also falls from £58 to £51 and attentive CTG appears less cost-effective (the associated probability of cost-effectiveness is reduced from 70 to 62%). Such a finding is intuitive because the base-case analysis modelled a reduction in intrapartum stillbirths as a consequence of the improved prognostic accuracy with the aid, but no change in compromise status. This in turn was associated with an increase in the number of severely compromised infants alive at two years (see Table 2) and thus a rise in the likely number of litigation claims submitted.

The various stage 2 analyses were also re-run including litigation costs. They showed that if severe compromise can be prevented for increasing proportions of the additional ‘at risk’ babies correctly identified by attentive CTG, then the mean cost savings and QALY gains begin to increase, along with the INMB, and cost-effectiveness. The dashed line in Fig. 3 plots the INMB for the base-case and each stage 2 analysis with litigation costs included.

Scaling up these data to a national level for England suggests that attentive CTG could correctly identify an additional 248 ‘at risk’ term babies. If severe compromise could be prevented in half of these babies, the model predicted a potential reduction in litigation costs of around £33.5 million. If prevented for all 248 additionally identified babies, predicted savings were around £67 million.

Supplementary Table S10 shows the base-case results were not greatly sensitive to changes in the price of attentive CTG. Analyses exploring further improvements to sensitivity and specificity however suggested that even small improvements to specificity e.g. from 0.90 to 0.91 and 0.92 have the potential to increase the base-case INMB from £58 to £78 and £98 respectively (see supplementary Tables S11 and S12). The associated probabilities of attentive CTG being cost-effective increase from 70 to 75% and 80% respectively.

## Discussion

The early health economic model presented in this paper has explored the potential cost-effectiveness of using a new computerised decision support aid alongside current practice to identify signs of fetal compromise during CTG monitored term labours. The base-case analysis incorporated improvements in alert accuracy considered achievable with version 3.0 of the attentive CTG tool, and suggested that even without reducing the risk of severe compromise in additional babies correctly identified as ‘at risk’ during labour, it has a 70% chance of being cost-effective at a willingness to pay threshold value of £25,000 per QALY. Interestingly, such results were driven by cost savings arising as a result of the small anticipated improvements in specificity, and without any reduction in sensitivity. With unnecessary labour alerts possibly affecting up to 13% of monitored women, increasing specificity from 0.87 to 0.90 would avoid many of these alerts, as well as the more costly, clinically risky, and traumatic emergency instrumental deliveries that follow. At a national level, data suggested a possible reduction of some 2,400 emergency C-sections across England (Table 2). It is important to stress that such cost savings are predicted to arise through better identification of women not requiring expedited delivery and not by impacting the care of women and babies who require intervention.

In contrast, and as the prevalence of severe compromise is low (0.4% in the John Radcliffe Hospital cohort) the larger improvements in alert sensitivity anticipated with attentive CTG (from 0.38 to 0.55), resulted in a seemingly small yet important increase in the number of compromised babies being correctly identified as ‘at risk’ during labour. An estimated 18 additional babies in the John Radcliffe Hospital cohort would be identified (almost 30% of the severely compromised babies without a previous alert). Analyses showed that cost-effectiveness results were sensitive to assumptions made about the impact that the alert and any expedited birth could have on reducing the risk of severe compromise for these additionally identified babies (the stage 2 analyses). Assuming severe compromise could be prevented for half or even all of these babies increased the likelihood of attentive CTG being cost-effective to 85% and 95% respectively (Fig. 4).

The shape of the cost-effectiveness acceptability curves in Fig. 4 is worthy of discussion. Showing a decline in the probability that attentive CTG is cost-effective with increasing levels of willingness to pay for an additional QALY, the curves reflect the location of the cloud of incremental cost and QALY pairs generated by the PSA, on the cost-effectiveness plane (see supplementary Figure S1). With the tool altering health outcomes for only a proportion of the already small number of severely compromised infants, the overall mean QALY gain is close to zero, and the confidence interval is wide on account of the limited data available to inform model parameters for this group of individuals. As a result, and given the projected cost savings, the cloud of cost/QALY pairs predominantly spans the south west and south east quadrants. When the maximum willingness to pay for a QALY is increased, the gradient of the line denoting this threshold increases and the proportion of points on the plane that fall to the right of this line, is reduced, and with it the likelihood of cost-effectiveness.

The perspective adopted for the study was that of the NHS in England and only delivery and infant healthcare costs and QALYs were included. In reality the consequences of improving the prognostic accuracy of labour monitoring will be far more wide reaching. For example, whilst the benefits of emergency C-section are well documented, the procedure is not without its risks. Alongside the acknowledged surgery related morbidity such as wound infection, haemorrhage, thrombosis, and future fertility problems, emergency C-sections have known associations with maternal post-traumatic stress, and with reductions in health-related quality of life, self-esteem, infant bonding, and breast-feeding [54, 55]. The prevention of a sizeable number of emergency C-sections without reducing the sensitivity with attentive CTG could therefore be expected to lead to improvements in maternal quality of life and further reductions in healthcare costs.

Additionally, the implications for families and wider society of raising a child with a severe disability are profound, with many studies reporting reduced parental employment, financial hardship, increased levels of stress, and poor mental health endured by parents and other family members [56–64]. Extra resources are also required for the provision of services such as education and social care and there is often a need for ongoing support into adulthood [65]. If attentive CTG can reduce the number of severely compromised babies through better prognostic accuracy and delivery management, then once again, there are likely to be further cost savings and health benefits for families and society as a whole. Thus one can hypothesise, that the cost-effectiveness results presented could be conservative in nature and that a wider study perspective could have demonstrated further cost savings and health gains from using attentive CTG.

Comparing the findings reported here with those of other studies evaluating the cost-effectiveness of computerised decision aids for identifying fetal compromise during labour is challenging for a number of reasons. Firstly, attentive CTG as modelled here is fundamentally different from previous systems developed. They predict an abnormal CTG by assessing ‘classic’ CTG patterns against established guidelines or clinician defined criteria on CTG interpretation and do not adjust risks for multiple confounding clinical factors and labour stage [1, 12, 13]. In contrast, attentive CTG is a data-driven tool trained on real outcomes, with predictions for the risk of severe compromise based upon prognostic models estimated using large patient-level datasets containing information from CTG traces, maternal, pregnancy, and labour characteristics and baby outcomes. It therefore facilitates individualised risk prediction and allows simulated sensitivity and specificity estimates, which are unavailable for clinical practice or previous computerised decision aids not based on data, and so preclude their modelling. The system will also incorporate aspects of artificial intelligence in the form of Deep Learning, and can learn from the raw CTG signals to further improve the accuracy of its predictions [17, 18].

Secondly, the work presented in this paper was not conducted for the purposes of generating definitive cost-effectiveness results. Rather, the aim of this early health economic modelling was to help guide the future research pathway for attentive CTG by exploring its potential to offer the NHS value for money and helping inform decisions around the investment of further resources into prospective evaluative assessments of its effectiveness and cost-effectiveness. Comparisons with definitive cost-effectiveness analyses in this area would not therefore be appropriate.

The study has a number of strengths, including the availability of a large patient-level dataset to inform many parameters for the model. Although these data came from a single NHS hospital, we were able to demonstrate that the model’s predictions of key events, when scaled up to a national level, were in line with those reported by other initiatives such as Each Baby Counts. A further strength is the secondary analysis including litigation costs. Maternity-related negligence is substantial and costs the NHS over £1bn per year. This work was able to predict that attentive CTG has the potential to reduce these costs alongside the costs of delivering babies. In the spirit of the exploratory early health economic model, we would caution against placing undue weight on the projected litigation cost savings per se. Their inclusion served to demonstrate improvements to the potential cost-effectiveness of attentive CTG, however it is acknowledged that a more rigorous investigation of these costs is required.

Of course this work is not without its limitations. Estimating the sensitivity and specificity of current practice for the model is problematic, as for some women with suspected fetal compromise, an interventional delivery may have altered their baby’s outcome. For example, amongst the women in the second row of column C in Table 1 who appear to have had a false positive alert with current practice (a labour alert but a non-severely compromised baby) there will be a proportion for whom an alert was correctly raised and a subsequent intervention prevented the severe compromise of their baby. The implication of this is that the false positive rate for current practice and thus the potential absolute benefits of attentive CTG in reducing this (for example through fewer ‘unnecessary’ emergency Caesarean sections) will be overestimated. The extent of this overestimation is difficult to quantify, however it is likely to be small. A 2017 Cochrane systematic review of historical trials comparing CTG with intermittent auscultation (12 trials with over 37,000 women) reported that overall, adverse infant outcomes were around one in 300 for perinatal deaths and one in 500 for infant seizures [6]. It also found that whilst CTG reduced seizure rates, it did not reduce perinatal deaths, cerebral palsy, or other measures of neonatal wellbeing. Secondly, CTG is known to be associated with high false positive rates and consequently unnecessary intervention [66]. The same systematic review for example reported that despite not having an impact upon perinatal mortality and other infants outcomes, CTG had led to a significant increase in Caesarean section rates (relative risk 1.63, 95% CI 1.29 to 2.07) [6]. When taken together, these findings make it probable that the majority of the women classified as false positive in Table 1 were indeed those for whom an unnecessary alert was raised. Whilst there will be some women in this group for whom an alert was correctly raised and an interventional delivery prevented harm to their baby, given the adverse event prevalence estimates and the infant outcome findings reported by the systematic review, such numbers will likely be small.

A further limitation is that the model is, as with any model, a simplification of a real-world process, and it was not possible to identify data for all parameters. For example, it was not possible to estimate the reduction in the risk of severe compromise with an expedited delivery following an accurate alert with attentive CTG. We employed scenario analyses to evaluate the impact of different thresholds of risk reductions on cost-effectiveness results. Finally, the patient level dataset used to inform parameter values for the model, included deliveries for the period 2013 to 2018. Practice has changed over recent years and intrapartum investigation and diagnosis of presumed fetal compromise has increased. As such specificity as estimated for the current practice arm of the model may be slightly higher than what is observed in current practice today.

## Conclusions

Based upon the early health economic model presented in this paper, it would appear that attentive CTG developed to help clinicians identify term babies at risk of severe compromise during a monitored labour, has the potential to offer value for money to the NHS. If the estimated prediction accuracy statistics for attentive CTG can be realised in practice, the model suggests that cost savings could potentially be realised by reducing unnecessary alerts and interventional deliveries, and that the likelihood of cost-effectiveness would be high, even if severe compromise could be avoided for only a proportion of the additional ‘at risk’ babies correctly identified. Research should now focus upon the design and conduct of prospective evaluative studies, with the aim of definitively assessing the effectiveness and cost-effectiveness of adding attentive CTG to routine care.

## Supplementary Information

Supplementary material 1

## Data Availability

The data supporting the estimation of key parameters for the model are available from the corresponding author upon reasonable request.

## Abbreviations

CTG: Cardiotocography
NHS: National Health Service
QALYs: Quality-adjusted life years
RCOG: Royal College of Obstetricians and Gynaecologists
EPR: Electronic patient record
NNU: Neonatal unit
PSA: Probabilistic sensitivity analysis
CEACs: Cost-effectiveness acceptability curves
INMB: Incremental net monetary benefit
