# Supplementary material for: Exploring the potential cost-effectiveness of a new computerised decision support tool for identifying fetal compromise during monitored term labours: an early health economic model
Source: Cost Eff Resour Alloc. 2024 Oct 7;22:72. doi: 10.1186/s12962-024-00580-x (PMC11460220; doi:10.1186/s12962-024-00580-x)
Supplement: Supplementary file 1 — Supplementary material 1 [file 12962_2024_580_MOESM1_ESM.docx]

**Supplementary file accompanying manuscript:**

**Exploring the potential cost-effectiveness of a new computerised decision support tool for identifying fetal compromise during monitored term labours: an early health economic model.**

Cost-effectiveness and Resource Allocation

Campbell HE1* (ORCID ID 0000-0003-2070-7794), Ratushnyak S1* (ORCID ID 0000-0001-7967-5112), Georgieva A2 (ORCID ID 0000-0002-5543-6683), Impey L3 (ORCID ID 0000-0002-4462-112X), Rivero-Arias O1† (ORCID ID 0000-0003-2233-6544).

*Joint first authors

1National Perinatal Epidemiology Unit, Nuffield Department of Population Health, University of Oxford, Oxford, UK.

2Oxford Labour Monitoring Group, Nuffield Department of Women’s and Reproductive Health, University of Oxford, Oxford, UK.

3Fetal Medicine Unit, John Radcliffe Hospital, Oxford, UK.

†Corresponding author

Assoc. Professor Oliver Rivero-Arias

National Perinatal Epidemiology Unit,

Nuffield Department of Population Health,

University of Oxford,

Oxford, UK.

Email: oliver.rivero@npeu.ox.ac.uk

**Table of contents**

[Definition of severe compromise used in the economic model 3](#_Toc178773312)

[Extended modelling methods 4](#_Toc178773313)

[Event probabilities 4](#_Toc178773314)

[Health-related quality of life 9](#_Toc178773315)

[Unit costs 10](#_Toc178773316)

[Modelling the impact of attentive CTG 18](#_Toc178773317)

[Base-case analysis 18](#_Toc178773318)

[Additional scenario analyses 20](#_Toc178773319)

[Results 21](#_Toc178773320)

[Reference list 32](#_Toc178773321)

# Definition of severe compromise used in the economic model

Table S1 below lists the Each Baby Counts outcomes that were used to define severe compromise within the model.

**Table S1 Outcomes used to define severe compromise within the early health economic model**

| *Definition of ‘Severely Compromised’ based on Each Baby Counts definition (1)* | All term babies (at least 37+0 completed weeks of gestation) born following labour who have one of the following outcomes:   - **Intrapartum stillbirth:** when the baby was thought to be alive at the start of labour but was born with no signs of life. - **Early neonatal death:** when the baby died within the first week of life (i.e. days 0–6) of any cause. - **Severe brain injury** diagnosed in the first 7 days of life, when the baby:   - was diagnosed with grade III hypoxic ischaemic encephalopathy (HIE) OR   - was therapeutically cooled (active cooling only) OR   - had decreased central tone AND was comatose AND had seizures of any kind. |
| --- | --- |

# Extended modelling methods

## Event probabilities

Table S2 below details the values and sources of the event probabilities used to populate the decision tree component of the early health economic model.

**Table S2 Values and sources of event probabilities used within the usual care arm of the decision tree**

| **Event Probability^a^** | **Value**  **(Distribution type; parameters)** | **Source^b^; Notes** |
| --- | --- | --- |
| ***Decision – support*** |  |  |
| Probability of an alert for PFC during a term, monitored labour^c^ | 0.132352  (Beta; α=3,022; β=19,811) | -- |
| ***Delivery outcomes*** |  |  |
| Probability of a live birth following an alert for PFC^c^ | 1.00000  (Beta; α=3,022; β=0) | -- |
| Probability of a live birth following no alert for PFC^c^ | 0.999748  (Beta; α=19,806; β=5) | -- |
| ***Delivery type (live birth only)^d^*** |  |  |
| Probability of a spontaneous delivery following an alert for PFC | 0  -- | Not observed |
| Probability of an assisted delivery following an alert for PFC | 0.616810  (Beta; α=1,864; β=1,158) | -- |
| Probability of a spontaneous delivery following no alert for PFC | 0.647279  (Dirichlet; α_1_=12,820; α_2_=4,242; α_3_=2,744) | -- |
| Probability of an assisted delivery following no alert for PFC | 0.214178  (Dirichlet; α_1_=12,820; α_2_=4,242; α_3_=2,744) | -- |
| ***Baby health outcomes*** |  |  |
| Probability of severe compromise following an alert for PFC and a spontaneous delivery^c^ | NA  -- | -- |
| Probability of severe compromise following an alert for PFC and an assisted delivery^c^ | 0.010193  (Beta; α=19; β=1,845) | -- |
| Probability of severe compromise following an alert for PFC and an emergency C-section delivery^c^ | 0.016408  (Beta; α=19; β=1,139) | -- |
| Probability of severe compromise following no alert for PFC and a spontaneous delivery^c^ | 0.002184  (Beta; α=28; β=12,792) | -- |
| Probability of severe compromise following no alert for PFC and an assisted delivery^c^ | 0.004715  (Beta; α=20; β=4,222) | -- |
| Probability of severe compromise following no alert for PFC and an emergency C-section delivery^c^ | 0.003644  (Beta; α=10; β=2,734) | -- |
| ***Neonatal outcomes before hospital discharge^e^*** |  |  |
| Probability of no NNU admission for severely compromised babies | 0.083333  (Dirichlet; α1=8; α2=67; α3=21) | -- |
| Probability of NNU admission and survival for severely compromised babies | 0.697917  (Dirichlet; α1=8; α2=67; α3=21) | -- |
| Probability of no NNU admission for not severely compromised babies^c^ | 0.914174  (Beta; α=20,781; β=1,951) | -- |
| ***Baby outcomes from hospital discharge up to Year 2*** |  |  |
| Probability of death in the two years following hospital discharge for a severely compromised baby | 0.024194  (Beta; α=3; β=121) | Assumed to be equal to the 18-month mortality rate for infants who were discharged after undergoing total body cooling for perinatal asphyxia at birth, in the TOBY trial (2). |
| Probability of death in the two years following hospital discharge for a not severely compromised baby | 0.000585  (Beta; α=336; β=573,496) | Estimated using number of deaths and population at risk during first two years of life, from the Child Mortality Statistics for England and Wales, 2020 (3). |

PFC presumed fetal compromise; NNU neonatal unit; NA not applicable

^a^Event probability headings below correspond to those in Figure 1 of the main paper.

^b^Unless otherwise stated, event probability was estimated using observed clinical event data from women who were CTG monitored during a term labour (gestational age at birth is at least 37+0 completed weeks) at the John Radcliffe Hospital in Oxford, between 2013 and 2018.

^c^The complement is given by the residual, for example the probability of no labour alert is given by 1 minus the probability of an alert.

^d^Probability of an emergency C-section delivery for each of the alert and no alert groups is not shown but is given by 1 minus the sum of the associated probabilities of spontaneous and assisted deliveries.

^e^Probability of a NNU admission and in-hospital baby death for the severely compromised group is not shown but is given by 1 minus the sum of the associated probabilities of no NNU admission and NNU admission and survival. Probability of a NNU admission and in-hospital baby death for not severely compromised baby group is equal zero. As a result, a beta distribution was used for neonatal outcomes before hospital discharge for non-severely compromised babies.

Table S3 below details the values and sources of the event probabilities used to populate the Markov part of the early health economic model.

As noted in the main paper, the group of none severely compromised children alive at two years and entering the Markov model were exclusively without neurodevelopmental disabilities. In contrast, the severely compromised group of children alive at two years included infants both with and without neurodevelopmental disabilities.

For the severely compromised group, we thus calculated the input parameters (annual mortality probabilities, health-related quality of life, costs) by taking weighted averages of parameters for disabled and non-disabled children. Weights were estimated using data from the TOBY trial on proportions of children who experienced perinatal asphyxia at birth, underwent therapeutic cooling, and were then diagnosed with neurodevelopmental disabilities at 18 months (41%) (2) and at seven years (32%) (4). For the Markov model, we assumed that the proportion of compromised babies diagnosed with neurodevelopmental disability in the TOBY trial at 18 months would be the same at two years. Additionally, we assumed a linear annual decrease in the disability proportion from two years up to seven years. Beyond this point, and in the absence of longer term data, it was assumed the proportion of severely compromised children with neurodevelopmental disability each year remained constant up to 18 years.

Annual probabilities of death for severely compromised children surviving beyond two years were thus estimated as a weighted average of annual mortality risks for children without neurodevelopment disability (informed by National lifetables for England) and annual mortality risks for children with disabilities estimated from survival data reported for over 3000 babies with cerebral palsy and a normal birth weight (5-7). National lifetable data were used to model survival for children in the Markov model who were not severely compromised at birth (6, 7).

Columns two and three of Table S3 below show the resulting annual mortality probabilities used within the Markov model for severely and not severely compromised children.

**Table S3 Transition probabilities, health state utilities, and healthcare costs used in the Markov component of the model (age 2 to 18 years)**

| **Year** | **Annual probability**  **of death**  **in SC^a^ (5, 6)** | **Annual probability**  **of death**  **in NSC (6)** | **Health-related quality of life weights**  **in SC (8, 9)** | **Health-related quality of life weights**  **in NSC (9)** | **Healthcare costs**  **in SC^b^ (10, 11)** | **Healthcare costs**  **in NSC (11)** |
| --- | --- | --- | --- | --- | --- | --- |
| **(1)** | **(2)** | **(3)** | **(4)** | **(5)** | **(6)** | **(7)** |
| **2-3** | 0.008550 | 0.000121 | 0.662 | 0.92 | £2,894 | £1,435 |
| **3-4** | 0.022317 | 0.000096 | 0.674 | 0.92 | £2,635 | £1,350 |
| **4-5** | 0.016778 | 0.000075 | 0.686 | 0.92 | £2,387 | £1,266 |
| **5-6** | 0.019920 | 0.000078 | 0.698 | 0.92 | £2,149 | £1,181 |
| **6-7** | 0.011653 | 0.000075 | 0.709 | 0.92 | £1,922 | £1,096 |
| **7-8** | 0.016754 | 0.000060 | 0.709 | 0.92 | £1,745 | £1,011 |
| **8-9** | 0.016961 | 0.000059 | 0.709 | 0.92 | £1,569 | £926 |
| **9-10** | 0.016409 | 0.000059 | 0.709 | 0.92 | £1,393 | £842 |
| **10-11** | 0.011020 | 0.000069 | 0.709 | 0.92 | £1,216 | £757 |
| **11-12** | 0.024385 | 0.000066 | 0.709 | 0.92 | £1,216 | £757 |
| **12-13** | 0.007073 | 0.000079 | 0.689 | 0.9 | £1,216 | £757 |
| **13-14** | 0.005451 | 0.000103 | 0.689 | 0.9 | £1,216 | £757 |
| **14-15** | 0.018318 | 0.000109 | 0.689 | 0.9 | £1,216 | £757 |
| **15-16** | 0.005893 | 0.000135 | 0.639 | 0.85 | £1,216 | £757 |
| **16-17** | 0.006016 | 0.000161 | 0.639 | 0.85 | £1,216 | £757 |
| **17-18** | 0.050293 | 0.000222 | 0.639 | 0.85 | £1,216 | £757 |

SC severely compromised; NSC non-severely compromised

^a^weighted average annual mortality risks across children with and without neurodevelopmental disability (see accompanying text for details).

^b^Excluding litigation costs. To include litigation costs an annual expected sum of £3,240 was added to healthcare costs to cover the annuitized portion of a payment (see section below on additional scenario analyses).

## Health-related quality of life

For the decision tree component of the economic model, utility scores (measured on a scale where 0 represents death and 1 full health) are unavailable for infants below the age of two years and so we assumed that the utility estimates we derived for the model for severely and non-severely compromised babies at two years of age (see row three of Table S3), would have applied to these same babies during the first two years of their lives. For non-severely compromised babies, utility scores were informed by age-specific population norms reported on the Health Utililties Index (HUI) questionnaire for Canadian children (9). For the group of severely compromised babies, a weighted average utility score was again estimated, acknowledging that a proportion of babies would be with / without neurodevelopmental disability (see above section on event probabilities for detail on how these proportions were estimated). The Canadian HUI population norms were used to inform utility for infants without disability and utility for infants with disability was assumed to be as reported by Petrou & Kupek and based on HUI questionnaire responses for a group of 178 UK children with cerebral palsy (8). When infants in the model died, their utility scores were set to zero.

Columns four and five of Table S3 show the annual utility weights assigned to severely and not severely compromised infants surviving each year in the Markov model. As detailed immediately above, utility for non-compromised infants was informed by age-specific HUI population norms for Canadian children, and for compromised infants was a weighted average of these HUI population norms and utility levels reported for a group of UK children with cerebral palsy (8, 9).

## Unit costs

Table S4 shows the values and sources of the unit costs used within the decision tree.

*In-hospital costs*

Included in in-hospital costs were costs for delivery type (spontaneous, assisted, or emergency C-section obtained from the 2020/21 National Schedule of NHS Costs), with a further distinction made by baby outcome. For example, costs for spontaneous deliveries without neonatal unit (NNU) admission were calculated using short-stay normal delivery codes whereas costs for spontaneous deliveries with NNU admissions were based upon normal delivery long-stay codes to reflect the likely accompanying longer maternal hospital stay. Additionally, delivery and investigation costs were included following an intrapartum stillbirth.

To estimate the costs associated with NNU admissions, length of stay data for infants born into the John Radcliffe cohort between 2013 and 2018 and admitted to the NNU were used to estimate the mean NNU stay for each pathway within the tree. Length of NNU stay was conditioned upon an infant’s survival status at discharge (survive / not survive), status at birth (severely / not severely compromised), delivery type, and labour alert status (see Table S4). In the absence of information on the level of care provided, to cost each mean NNU stay for babies who survived to hospital discharge, we applied a weighted average of the per diem costs for Neonatal Critical Care - currency codes XA01Z (Intensive Care), XA02Z (High Dependency), XA03Z (Special Care, without External Carer), XA04Z (Special Care, with External Carer), XA05Z (Normal Care) from the 2020/21 National Schedule of NHS Costs - £1,105 (12). For babies who died whilst in the NNU, we costed the duration of their stay at an intensive care level of provision (£1,810 - Neonatal Critical Care currency code XA01Z (Intensive Care), National Schedule of NHS Costs, 2020/2021) (12) and included the cost of a post-mortem (see Table S4).

*Costs post-discharge to 2 years*

Of the babies surviving beyond hospital discharge and out to 2 years, those who were not severely compromised and had not required NNU care were assumed to undergo the regular routine health check-ups and vaccinations as provided by the National Health Service in England, as well as to have primary care consultations and hospital stays as reported by Cecil et al. in their study of healthcare contacts for a population-based birth cohort of UK children (13-15). Unit costs for these contacts out to two years were obtained from the National Schedule of NHS Costs, 2020/2021 and Unit Costs of Health and Social Care 2021 (12, 16).

Healthcare costs for babies who survived out to two years and who had not been severely compromised but had required NNU care, would likely be higher than for non-compromised babies who had not required NNU care. For these babies we therefore assumed their healthcare contacts during the first two years would be similar to those observed for babies in the cooling group (n=102) of the TOBY trial who had experienced difficulties at birth but who were without neurodevelopmental disability (17). Costs reported for these babies during the first 12 months following birth and then between 12 and 18 months were inflated to 2020/2021 prices using the NHS cost inflation index (16). We assumed costs between 12 and 18 months in the TOBY study would also be representative of those incurred between 18 and 24 months.

To estimate healthcare costs for babies who survived out to two years after having been severely compromised at birth, we used data from the TOBY trial to determine the proportions that would and would not be suffering with neurodevelopmental disability (as described above in the section on event probabilities)(2). A weighted average cost was then estimated using data reported by the TOBY trial on infant healthcare costs in the cooling group (n=102) incurred during the first 12 months following birth and then between 12 and 18 months. For disabled babies, costs were assumed to be as for those observed for TOBY trial babies with neurological disabilities, and for those without disability, costs used were those for TOBY trial babies without neurological impairment (17). Costs were again inflated to 2020/2021 prices and as detailed above we assumed costs between 12 and 18 months in the TOBY study would also be representative of those incurred between 18 and 24 months.

For babies not severely compromised at birth and not requiring NNU care but who subsequently died between discharge and two years, we used a survival-based approach to costing, partitioning the two-year follow-up period into intervals with the probability of survival for each interval estimated using child and infant mortality statistics for England and Wales (3). These probabilities were then used to weight the estimated healthcare costs for a healthy baby (as described above for healthy babies surviving to two years) (13-15) falling within each time interval before these costs were summed to generate an expected cost for the two-year period. To this figure we also added the cost of an emergency ambulance transfer to hospital taken from the National Schedule of NHS Costs and the cost of a post-mortem investigation reported by Campbell et al. and inflated to 2020/2021 prices (12, 16, 18).

For babies severely compromised at birth and / or requiring NNU care, but who later died following hospital discharge, healthcare costs to two years were based upon those of infants within the TOBY trial, who died following hospital discharge (17) . These costs were inflated to 2020/2021 prices using the NHS cost inflation index (16). The costs of emergency transportation to hospital and a post-mortem were again included (12, 18).

**Table S4 Unit costs used within the decision tree component of the model**

| **Resource Use item** | **Unit Cost^a^**  **20/21 UK£** | **Source; Notes** |
| --- | --- | --- |
| ***Delivery type (live birth only)*** |  |  |
| Spontaneous delivery, baby not admitted to NNU and survives | £2,402 | Weighted average of Normal Delivery codes for Non-Elective Short Stay^b^, National Schedule of NHS Costs, 2020/2021 (12). |
| Spontaneous delivery, baby severely compromised, is admitted to NNU and survives | £4,938 | Weighted average of Normal Delivery codes for Non-Elective Long Stay^b^, National Schedule of NHS Costs, 2020/2021 (12). |
| Spontaneous delivery, baby severely compromised, is admitted to NNU and dies OR baby not severely compromised, is admitted to NNU and survives | £3,557 | Weighted average of Normal Delivery codes for Non-Elective Short and Long Stay^b^, National Schedule of NHS Costs, 2020/2021 (12). |
| Assisted delivery, baby not admitted to NNU and survives | £3,032 | Weighted average of Assisted Delivery codes for Non-Elective Short Stay^c^, National Schedule of NHS Costs, 2020/2021 (12). |
| Assisted delivery, baby severely compromised, is admitted to NNU and survives | £6,250 | Weighted average of Assisted Delivery codes for Non Elective Long Stay^c^, National Schedule of NHS Costs, 2020/2021 (12). |
| Assisted delivery, baby severely compromised, is admitted to NNU and dies OR baby not severely compromised, is admitted to NNU and survives | £5,421 | Weighted average of Assisted Delivery codes for Non-Elective Short and Long Stay^c^, National Schedule of NHS Costs, 2020/2021 (12). |
| Emergency C-section, baby severely compromised, is admitted to NNU and survives | £8,060 | Weighted average of Emergency Caesarean Section codes for Non-Elective Long Stay^d^, National Schedule of NHS Costs, 2020/2021 (12). |
| Emergency C-section delivery, baby severely compromised, is admitted to NNU and dies OR baby not severely compromised, is admitted to NNU and survives, OR baby does not require NNU admission. | £7,584 | Weighted average of Emergency Caesarean Section codes for Non-Elective Short and Long Stay^d^, National Schedule of NHS Costs, 2020/2021 (12). |
|  |  |  |
| Stillbirth (intrapartum only) | £3,695 | Weighted average cost of Normal, Assisted, and Emergency Caesarean Section codes for Non-Elective Short Stay, National Schedule of NHS Costs, 2020/2021 (12) plus post-mortem cost from Campbell et al. 2018 (18) inflated to 2020/2021 prices (16). |
| ***Neonatal unit care (NNU) following delivery*** |  |  |
| *Babies surviving NNU* |  |  |
| Baby survives NNU admission after alert for PFC, assisted delivery, and severe compromise | £8,750  (Gamma,  α=1.717, λ=0.0002) | JRH EPR data 2013-2018, National Schedule of NHS Costs, 2020/2021 (12). |
| Baby survives NNU admission after alert for PFC, assisted delivery, and no severe compromise | £2,671  (Gamma,  α=0.38072, λ=0.00014) | JRH EPR data 2013-2018, National Schedule of NHS Costs, 2020/2021 (12). |
| Baby survives NNU admission after alert for PFC, emergency C-section, and severe compromise | £13,770  (Gamma,  α=1.75035, λ=0.00013) | JRH EPR data 2013-2018, National Schedule of NHS Costs, 2020/2021 (12). |
| Baby survives NNU admission after alert for PFC, emergency C-section, and no severe compromise | £4,467  (Gamma,  α=0.47327, λ=0.00011) | JRH EPR data 2013-2018, National Schedule of NHS Costs, 2020/2021 (12). |
| Baby survives NNU admission after no alert for PFC, spontaneous delivery, and severe compromise | £12,941  (Gamma,  α=4.14624, λ=0.00032) | JRH EPR data 2013-2018, National Schedule of NHS Costs, 2020/2021 (12). |
| Baby survives NNU admission after no alert for PFC, spontaneous delivery, and no severe compromise | £3,592  (Gamma,  α=0.33883, λ=0.00009) | JRH EPR data 2013-2018, National Schedule of NHS Costs, 2020/2021 (12). |
| Baby survives NNU admission after no alert for PFC, assisted delivery, and severe compromise | £8,566  (Gamma,  α=3.261, λ=0.00038) | JRH EPR data 2013-2018, National Schedule of NHS Costs, 2020/2021 (12). |
| Baby survives NNU admission after no alert for PFC, assisted delivery, and no severe compromise | £2,349  (Gamma,  α=0.28223, λ=0.00012) | JRH EPR data 2013-2018, National Schedule of NHS Costs, 2020/2021 (12). |
| Baby survives NNU admission after no alert for PFC, emergency C-section, and severe compromise | £12,803  (Gamma,  α=2.25812, λ=0.00018) | JRH EPR data 2013-2018, National Schedule of NHS Costs, 2020/2021 (12). |
| Baby survives NNU admission after no alert for PFC, emergency C-section, and no severe compromise | £2,671  (Gamma,  α=0.40623, λ=0.00015) | JRH EPR data 2013-2018, National Schedule of NHS Costs, 2020/2021 (12). |
| ***Neonatal unit care (NNU) following delivery*** |  |  |
| *Babies dying in NNU* |  |  |
| Baby death during NNU admission after alert for PFC, assisted delivery, and severe compromise | £6,387  (Gamma,  α=3.75579, λ=0.00059) | JRH EPR data 2013-2018, National Schedule of NHS Costs, 2020/2021 (12), Campbell et al. 2018 (18) inflated to 2020/2021 prices (16). |
| Baby death during NNU admission after alert for PFC, emergency C-section, and severe compromise | £8,649  (Gamma,  α=5.32486, λ=0.00062) | JRH EPR data 2013-2018, National Schedule of NHS Costs, 2020/2021 (12), Campbell et al. 2018 (18) inflated to 2020/2021 prices (16). |
| Baby death during NNU admission after no alert for PFC, spontaneous delivery, and severe compromise | £10,836  (Gamma,  α=0.46078, λ=0.00004) | JRH EPR data 2013-2018, National Schedule of NHS Costs, 2020/2021 (12), Campbell et al. 2018 (18) inflated to 2020/2021 prices (16). |
| Baby death during NNU admission after no alert for PFC, assisted delivery, and severe compromise | £5,482  (Gamma,  α=8.65817, λ=0.00158) | JRH EPR data 2013-2018, National Schedule of NHS Costs, 2020/2021 (12), Campbell et al. 2018 (18) inflated to 2020/2021 prices (16). |
| Baby death during NNU admission after no alert for PFC, emergency C-section, and severe compromise | £6,538  (Gamma,  α=3.93523, λ=0.0006) | JRH EPR data 2013-2018, National Schedule of NHS Costs, 2020/2021 (12), Campbell et al. 2018 (18) inflated to 2020/2021 prices (16). |
| ***Infant costs from hospital discharge to Year 2*** |  |  |
| *Babies surviving to Year 2* |  |  |
| Baby survived, no admission to the NNU, not severely compromised | £1,515 | NHS - Your baby's health and development reviews (14), Cecil et al. 2018 (15). National Schedule of NHS Costs, 2020/2021, Jones and Burns, 2021 (12, 16). |
| Baby survived, admission to the NNU, not severely compromised | £3,758  (Gamma,  α=11.2361,  λ=0.00299) | Regier et al 2010 (17), Jones and Burns, 2021 (16). |
| Baby survived, severely compromised | £5,407  (Gamma,  α=14.3875,  λ=0.00266) | Regier et al 2010 (17), Jones and Burns, 2021 (16). |
| ***Infant costs from hospital discharge to Year 2*** |  |  |
| *Babies dying before Year 2* |  |  |
| Baby death following hospital discharge, no admission to the NNU, not severely compromised | £2,189 | NHS - Your baby's health and development reviews (14), Cecil et al. 2018 (15), National Schedule of NHS Costs, 2020/2021 [12], Jones and Burns, 2021 (16), NHS Child and Infant Mortality Statistics for England and Wales, 2020 (3), Campbell et al. 2018 (18). |
| Baby death following hospital discharge, severely compromised OR not severely compromised but admitted to NNU. | £3,444  (Gamma,  α=5.03064, λ=0.00146) | Regier et al 2010 (17), Jones and Burns, 2021 (16) and adjusted to 2020/2021 prices using the NHS cost inflation index (16), National Schedule of NHS Costs, 2020/2021 (12), Campbell et al. 2018 (18). |

PFC presumed fetal compromise; NNU neonatal unit; NHS National Health Service; JRH John Radcliffe Hospital; EPR electronic patient record

^a^Where available, distribution type and parameters are given

^b^Normal delivery codes are NZ30A, NZ30B, NZ30C, NZ31A, NZ31B, NZ31C, NZ32A, NZ32B, NZ32C, NZ33A, NZ33B, NZ33C, NZ34A, NZ34B, and NZ34C

^c^Assisted delivery codes are NZ40A, NZ40B, NZ40C, NZ41A, NZ41B, NZ41C, NZ42A, NZ42B, NZ42C, NZ43A, NZ43B, NZ43C, NZ44A, NZ44B, and NZ44C

^d^Emergency C-section delivery codes are NZ51A, NZ51B, and NZ51C

Columns six and seven of Table S3 show the annual healthcare costs estimated to be incurred by severely compromised and non-compromised children surviving each year in the Markov model. For both groups, the relevant annual healthcare costs estimated for survivors at two years (see section above on post-discharge costs to two years) were assumed to decline linearly year on year until they reached the estimates at 11 years reported by Petrou and colleagues in their study looking at neurodevelopmental disability and economic outcomes (10). For non-compromised babies, 11-year costs were those reported by Petrou et al. for a cohort of babies without severe impairment (n=135) and for compromised babies were a weighted average of the costs reported for babies without severe impairment (n=135) and with severe impairment (n=22). Beyond 11 years and in the absence of longer-term data, annual costs were assumed to remain constant for each group (see Table S3).

# Modelling the impact of attentive CTG

## Base-case analysis

Table S5 below shows delivery outcomes observed within the John Radcliffe EPR dataset for the four mutually exclusive combinations of alert status and baby compromise status. The cell letterings used in the table correspond to those used in Table 1 of the main paper. Improvements in sensitivity with attentive CTG see women move from cell B up to cell A, and the risk of intrapartum stillbirth decrease from 8% to 0%. The risk of neonatal death remains unchanged. Improvements in specificity see women move from cell C to cell D, but with no stillbirth and neonatal deaths observed amongst these babies, such changes result only in changes to delivery types as illustrated in Table S6 below.

**Table S5 Number (proportion) of stillbirths and neonatal deaths observed by labour alert status and baby compromised status in the John Radcliffe Hospital EPR dataset of monitored term deliveries from 2013-2018**

|  | **Severely compromised babies**  **n (cell proportion)** | | | **Non-severely compromised babies**  **n (cell proportion)** | | |
| --- | --- | --- | --- | --- | --- | --- |
|  |  | **Intrapartum Stillbirths** | **Neonatal deaths** |  | **Intrapartum Stillbirths** | **Neonatal deaths** |
| **Labour alert for PFC** | **Cell A** | 0/38 (0.00) | 8/38 (0.21) | **Cell C** | 0/2,984 (0.00) | 0/2,984 (0.00) |
| **No labour alerts for PFC** | **Cell B** | 5/63 (0.08) | 13/63 (0.21) | **Cell D** | 0/19,748 (0.00) | 0/19,748 (0.00) |

PFC presumed fetal compromise

Arrows show direction of movements under stage 1 modelling of improvements in sensitivity (B to A) and specificity (C to D) with attentive CTG.

Table S6 below shows observed delivery types within the John Radcliffe dataset for the four mutually exclusive combinations of alert status and baby compromise status. Again the cell letterings used in the table correspond to those used in Table 1 of the main paper. For women in cell B who delivered a severely compromised baby but had not received an alert, the probabilities of a spontaneous, assisted or emergency C-section delivery were 48%, 34%, and 17% respectively. For the proportion of these women who, with attentive CTG, now receive and alert and move to Cell A, there is now a 0% chance of having a spontaneous delivery, a 50% chance of an assisted delivery and a 50% chance of an emergency C-section.

Improvements in specificity move women from Cell C to Cell D. Such women would initially have had a 0% chance of a spontaneous delivery, a 62% chance of an assisted delivery, and a 38% chance of an emergency C-section. Now the likelihood of a spontaneous delivery increases to 65% and the likelihood of an assisted delivery and emergency C-section decreases to 21% and 14% respectively.

**Table S6 Number and proportion of delivery types observed by labour alert status and baby compromised status in the John Radcliffe Hospital EPR dataset of monitored term deliveries from 2013-2018**

|  | | **Infant severely compromised**  **% within live births** | | **Infant not severely compromised**  **% within live births** | |
| --- | --- | --- | --- | --- | --- |
| **Labour alert for PFC** | Spontaneous delivery | **Cell A** | 0% (0/38) | **Cell C** | 0% (0/2,984) |
|  | Assisted delivery |  | 50% (19/38) |  | 62% (1,845/2,984) |
|  | Emergency C-section |  | 50% (19/38) |  | 38% (1,139/2,984) |
| **No labour alert for PFC** | Spontaneous delivery | **Cell B** | 48% (28/58) | **Cell D** | 65% (12,792/19,748) |
|  | Assisted delivery |  | 34% (20/58) |  | 21% (4,222/19,748) |
|  | Emergency C-section |  | 17% (10/58) |  | 14% (2,734/19,748) |

PFC presumed fetal compromise

Arrows show direction of movements under stage 1 modelling of improvements in sensitivity (B to A) and specificity (C to D) with attentive CTG.

## Additional scenario analyses

*Litigations Costs*

We conducted an analysis to estimate and incorporate further costs borne by the NHS as a result of legal claims for negligence made by parents of babies who suffer severe compromise at birth. To inform this analysis, we used data reported by the National Neonatal Research Database on the annual number of term babies suffering brain injury at birth in England, and using data reported by NHS Resolution on the annual numbers of claims made against the NHS for cerebral palsy and neonatal brain damage, we ascertained that around 10% of parents of severely compromised term babies would likely submit a claim (19, 20). NHS Resolution data further indicated that around 27% of these claims were successful / had a high probability of success, suggesting that overall, 2.7% of parents of children severely compromised at birth would receive compensation.(20) We utilised settlement amounts reported by solicitors firms together with information from NHS Resolution on the current average financial reserve for a cerebral palsy claim, to estimate a successful claim value of around £10million (20). As data suggested around 40% of the compensation would be paid as a lump sum, an expected cost of £104,348 was assigned to severely compromised individuals in the model at two years and the remaining expected portion annuitized. We implemented litigation costs within the model, in this manner, assuming a life expectancy of some 50 years for the child.

*Other scenarios*

Other scenarios modelled included a 25% increase and decrease to the cost of attentive CTG used within the model (£13.22) and additional improvements to sensitivity and specificity that may be achievable with further evolution of the prediction modelling software.

# Results

**Table S7 External validity of model outputs for current practice for monitored term births (gestational age at birth is at least 37+0 completed weeks)**

| **Clinical outcomes** | **Model output**  **current practice N (%)** | **NHS maternity statistics**  **(2021) N (%) (3, 21, 22)** | **MBRRACE-UK**  **(2014-2019) N (%) (23)** | **Each Baby Counts**  **(2015-2018) N (%) (1)** |
| --- | --- | --- | --- | --- |
| Emergency C-sections^a^ | 56,273  (17.16%) | 65,004  (11.85%) |  |  |
| Stillbirths | 72^b^  (0.02%) | 608  (0.11%) | 5,698  (0.14%) | 501^b^  (0.02%) |
| Neonatal deaths^a^ | 303  (0.09%) | 266  (0.05%) | 2,769  (0.07%) |  |
| Severely compromised babies at birth | 1,457  (0.44%) |  |  | 4,537  (0.16%) |
| Live births | 327,904  (99.56%) | 548,327  (99.89%) | 4,104,215  (99.86%) | 2,751,529  (99.98%) |
| All births | 329,361  monitored births | 548,935  (100%) | 4,109,913  (100%) | 2,752,030  (100%) |

NHS National Health Service; MBRRACE Mothers and Babies: Reducing Risk through Audits and Confidential Enquiries; C-section Caesarean section

^a^Among live births

^b^Intrapartum

**Table S8 Stage 2 analyses - predicted clinical outcomes with attentive CTG and current practice alone for an annual cohort of 329,361 monitored term births in England^a^**

| **Clinical outcomes** | **Current practice**  **N (SE)** | **Attentive CTG**  **N (SE)** | **Mean difference**  **(95% confidence interval)** | **% change** |
| --- | --- | --- | --- | --- |
| ***Stage 2 analysis with a 25% risk reduction of severe compromise for additional babies correctly identified as at risk by attentive CTG*** | | | | |
| Alerts for presumed fetal compromise | 43,591 (736) | 33,589 (648) | -10,002  (-11,931 to -8,072) | -22.94% |
| Emergency C-sections | 56,273 (821) | 53,869 (809) | -2,405  (-4,670 to -140) | -4.27% |
| Intrapartum stillbirths | 72 (32) | 51 (27) | -21  (-103 to 62) | -28.64% |
| Neonatal deaths | 303 (66) | 290 (65) | -13  (-193 to 167) | -4.19% |
| Severely compromised babies alive at 2 years | 1,058 (123) | 1,026 (122) | -32  (-366 to 303) | -3.00% |
| All babies alive at 2 years | 328,768 (76) | 328,802 (72) | +34  (-164 to 232) | +0.01% |
| ***Stage 2 analysis with a 50% risk reduction of severe compromise for additional babies correctly identified as at risk by attentive CTG*** | | | | |
| Alerts for presumed fetal compromise | 43,591 (736) | 33,589 (648) | -10,002  (-11,931 to -8,072) | -22.94% |
| Emergency C-sections | 56,273 (821) | 53,861 (809) | -2,412  (-4,678 to -147) | -4.29% |
| Intrapartum stillbirths | 72 (32) | 51 (27) | -21  (-103 to 62) | -28.71% |
| Neonatal deaths | 303 (66) | 276 (63) | -26  (-205 to 152) | -8.70% |
| Severely compromised babies alive at 2 years | 1,058 (123) | 976 (119) | -82  (-412 to 248) | -7.73% |
| All babies alive at 2 years | 328,768 (76) | 328,817 (71) | +49  (-148 to 246) | +0.01% |
| ***Stage 2 analysis with a 75% risk reduction of severe compromise for additional babies correctly identified as at risk by attentive CTG*** | | | | |
| Alerts for presumed fetal compromise | 43,591 (736) | 33,589 (648) | -10,002  (-11,931 to -8,072) | -22.94% |
| Emergency C-sections | 56,273 (821) | 53,853 (809) | -2,420  (-4,685 to -155) | -4.30% |
| Intrapartum stillbirths | 72 (32) | 51 (27) | -21  (-103 to 62) | -28.67% |
| Neonatal deaths | 303 (66) | 263 (62) | -40  (-217 to 136) | -13.26% |
| Severely compromised babies alive at 2 years | 1,058 (123) | 926 (116) | -132  (-458 to 195) | -12.45% |
| All babies alive at 2 years | 328,768 (76) | 328,832 (69) | +64  (-131 to 259) | +0.02% |
| ***Stage 2 analysis with a 100% risk reduction of severe compromise for additional babies correctly identified as at risk by attentive CTG*** | | | | |
| Alerts for presumed fetal compromise | 43,591 (736) | 33,589 (648) | -10,002  (-11,931 to -8,072) | -22.94% |
| Emergency C-sections | 56,273 (821) | 53,844 (809) | -2,429  (-4,696 to -163) | -4.32% |
| Intrapartum stillbirths | 72 (32) | 51 (27) | -21  (-103 to 62) | -28.60% |
| Neonatal deaths | 303 (66) | 249 (60) | -54  (-228 to 121) | -17.78% |
| Severely compromised babies alive at 2 years | 1,058 (123) | 876 (113) | -182  (-504 to 141) | -17.17% |
| All babies alive at 2 years | 328,768 (76) | 328,847 (68) | +79  (-114 to 272) | +0.02% |

SE standard error; CTG cardiotocography; C-section Caesarean section

^a^Based on NHS maternity statistics, there were 548,935 births in England in 2021 and the model assumes that 60% of all deliveries annually are monitored in England (21).

**Table S9 Stage 2 analyses - mean (SE) per baby 18-year costs, QALYs and cost-effectiveness of attentive CTG compared with current practice**

| **Comparators** | **Mean (SE)**  **cost discounted** | **Mean cost difference**  **(95% confidence interval)** | **Mean (SE) QALYs**  **discounted** | **Mean QALY difference**  **(95% confidence interval)** | **Net monetary benefit (SE)** |
| --- | --- | --- | --- | --- | --- |
| ***Stage 2 analysis with a 25% risk reduction of severe compromise for additional babies correctly identified as at risk by attentive CTG*** | | | | | |
| Current practice | £16,697  (£327) | --- | 12.00139  (0.00311) | --- | £283,338  (£337) |
| Attentive CTG | £16,647  (£328) | -£49  (-£112 to £14) | 12.00299  (0.00301) | 0.00161  (-0.00671 to 0.00992) | £283,427  (£337) |
| ***Incremental net monetary benefit (95% confidence interval)*** | | | | | **£89**  **(-£138 to £317)** |
| ***Stage 2 analysis with a 50% risk reduction of severe compromise for additional babies correctly identified as at risk by attentive CTG*** | | | | | |
| Current practice | £16,697  (£327) | --- | 12.00139  (0.00311) | --- | £283,338  (£337) |
| Attentive CTG | £16,644  (£328) | -£52  (-£115 to £10) | 12.00411  (0.00295) | 0.00273  (-0.00551 to 0.01096) | £283,459  (£336) |
| ***Incremental net monetary benefit (95% confidence interval)*** | | | | | **£121**  **(-£104 to £346)** |
| ***Stage 2 analysis with a 75% risk reduction of severe compromise for additional babies correctly identified as at risk by attentive CTG*** | | | | | |
| Current practice | £16,697  (£327) | --- | 12.00139  (0.00311) | --- | £283,338  (£337) |
| Attentive CTG | £16,641  (£328) | -£56  (-£118 to £7) | 12.00523  (0.00289) | 0.00384  (-0.00432 to 0.01200) | £283,490  (£336) |
| ***Incremental net monetary benefit (95% confidence interval)*** | | | | | **£152**  **(-£71 to £375)** |
| ***Stage 2 analysis with a 100% risk reduction of severe compromise for additional babies correctly identified as at risk by attentive CTG*** | | | | | |
| Current practice | £16,697  (£327) | --- | 12.00139  (0.00311) | --- | £283,338  (£337) |
| Attentive CTG | £16,638  (£328) | -£59  (-£121 to £3) | 12.00635  (0.00282) | 0.00496  (-0.00310 to 0.01302) | £283,521  (£335) |
| ***Incremental net monetary benefit (95% confidence interval)*** | | | | | **£183**  **(-£38 to £403)** |

SE standard error; CTG cardiotocography

**Table S10 Mean (SE) per baby 18-year costs, QALYs and cost-effectiveness of attentive CTG compared with current practice** **for different costs of the aid**

| **Comparators** | **Mean (SE)**  **cost discounted** | **Mean cost difference**  **(95% confidence interval)** | **Mean (SE) QALYs**  **discounted** | **Mean QALY difference**  **(95% confidence interval)** | **Net monetary benefit (SE)** |
| --- | --- | --- | --- | --- | --- |
| ***Scenario analysis with a 25% decrease in the base-case attentive CTG price of £13.22*** | | | | | |
| Current practice | £16,697  (£327) | --- | 12.00139  (0.00311) | --- | £283,338  (£337) |
| Attentive CTG | £16,647  (£328) | -£49  (-£113 to £14) | 12.00188  (0.00308) | 0.00049  (-0.00791 to 0.00889) | £283,400  (£337) |
| ***Incremental net monetary benefit (95% confidence interval)*** | | | | | **£62**  **(-£168 to £291)** |
| ***Scenario analysis with a 25% increase in the base-case attentive CTG price of £13.22*** | | | | | |
| Current practice | £16,697  (£327) | --- | 12.00139  (0.00311) | --- | £283,338  (£337) |
| Attentive CTG | £16,654  (£328) | -£43  (-£106 to £20) | 12.00188  (0.00308) | 0.00049  (-0.00791 to 0.00889) | £ 283,393  (£337) |
| ***Incremental net monetary benefit (95% confidence interval)*** | | | | | **£55**  **(-£175 to £284)** |

SE standard error; CTG cardiotocography

**Table S11 Predicted classifications and numbers of alerts following potential improvements in alert sensitivity and specificity with attentive CTG for the base-case (Stage 1) and scenario analyses (across various sensitivity and specificity values)**

|  | **(Alert & SC)**  **(A)** | **(No Alert & SC)**  **(B)** | **Total SC**  **(A+B)** | **Sensitivity**  **(A/(A+B))** | **(Alert & NSC)**  **(C)** | **(No Alert & NSC)**  **(D)** | **Total NSC**  **(C+D)** | **Specificity**  **(D/(C+D))** | **Total Alerts**  **(A+C)** |
| --- | --- | --- | --- | --- | --- | --- | --- | --- | --- |
| **Stage 1^a^** |  |  |  |  |  |  |  |  |  |
| Base-case | 56 | 45 | 101 | **0.55** | 2,273 | 20,459 | 22,732 | **0.90** | 2,329 |
| **Scenario analysis^b^** |  |  |  |  |  |  |  |  |  |
| Scenario 1 | 58 | 43 | 101 | **0.57** | 2,273 | 20,459 | 22,732 | **0.90** | 2,331 |
| Scenario 2 | 61 | 40 | 101 | **0.60** | 2,273 | 20,459 | 22,732 | **0.90** | 2,334 |
| Scenario 3 | 56 | 45 | 101 | **0.55** | 2,046 | 20,686 | 22,732 | **0.91** | 2,102 |
| Scenario 4 | 58 | 43 | 101 | **0.57** | 2,046 | 20,686 | 22,732 | **0.91** | 2,104 |
| Scenario 5 | 61 | 40 | 101 | **0.60** | 2,046 | 20,686 | 22,732 | **0.91** | 2,107 |
| Scenario 6 | 56 | 45 | 101 | **0.55** | 1,819 | 20,913 | 22,732 | **0.92** | 1,875 |
| Scenario 7 | 58 | 43 | 101 | **0.57** | 1,819 | 20,913 | 22,732 | **0.92** | 1,877 |
| Scenario 8 | 61 | 40 | 101 | **0.60** | 1,819 | 20,913 | 22,732 | **0.92** | 1,880 |

SC severely compromised; NSC non-severely compromised

^a^The improved sensitivity with attentive CTG enables the identification of more compromised babies during labour, thus leading to altered delivery types and delivery outcomes (see Tables S5 and S6) but does not alter the overall number of severely compromised babies.

^b^The sensitivity and specificity values associated with each scenario can be seen in columns headed ‘Sensitivity’ and ‘Specificity’

**Table S12 Scenario analyses (across various sensitivity and specificity values) - mean (SE) per baby 18-year costs, QALYs and cost-effectiveness of attentive CTG compared with current practice**

| **Comparators** | **Mean (SE)**  **cost discounted** | **Mean cost difference**  **(95% confidence interval)** | **Mean (SE) QALYs**  **discounted** | **Mean QALY difference**  **(95% confidence interval)** | **Net monetary benefit (SE)** |
| --- | --- | --- | --- | --- | --- |
| ***Scenario 1 (sensitivity 0.57 and specificity 0.90)*** | | | | | |
| Current practice | £16,697  (£327) | --- | 12.00139  (0.00311) | --- | £283,338  (£337) |
| Attentive CTG | £16,651  (£328) | -£46  (-£109 to £18) | 12.00293  (0.00307) | 0.00054  (-0.00784 to 0.00892) | £283,397  (£337) |
| ***Incremental net monetary benefit (95% confidence interval)*** | | | | | **£59**  **(-£170 to £289)** |
| ***Scenario 2 (sensitivity 0.60 and specificity 0.90)*** | | | | | |
| Current practice | £16,697  (£327) | --- | 12.00139  (0.00311) | --- | £283,338  (£337) |
| Attentive CTG | £16,652  (£328) | -£45  (-£109 to £18) | 12.00201  (0.00306) | 0.00062  (-0.00774 to 0.00898) | £283,399  (£337) |
| ***Incremental net monetary benefit (95% confidence interval)*** | | | | | **£61**  **(-£168 to £290)** |
| ***Scenario 3 (sensitivity 0.55 and specificity 0.91)*** | | | | | |
| Current practice | £16,697  (£327) | --- | 12.00139  (0.00311) | --- | £283,338  (£337) |
| Attentive CTG | £16,631  (£328) | -£66  (-£132 to £0) | 12.00188  (0.00308) | 0.00049  (-0.00791 to 0.00889) | £283,416  (£337) |
| ***Incremental net monetary benefit (95% confidence interval)*** | | | | | **£78**  **(-£152 to £308)** |
| ***Scenario 4 (sensitivity 0.57 and specificity 0.91)*** | | | | | |
| Current practice | £16,697  (£327) | --- | 12.00139  (0.00311) | --- | £283,338  (£337) |
| Attentive CTG | £16,631  (£328) | -£66  (-£131 to £0) | 12.00193  (0.00307) | 0.00054  (-0.00784 to 0.00892) | £283,417  (£337) |
| ***Incremental net monetary benefit (95% confidence interval)*** | | | | | **£79**  **(-£151 to £309)** |
| ***Scenario 5 (sensitivity 0.60 and specificity 0.91)*** | | | | | |
| Current practice | £16,697  (£327) | --- | 12.00139  (0.00311) | --- | £283,338  (£337) |
| Attentive CTG | £16,632  (£328) | -£65  (-£131 to £1) | 12.00201  (0.00306) | 0.00162  (-0.00774 to 0.00898) | £283,418  (£337) |
| ***Incremental net monetary benefit (95% confidence interval)*** | | | | | **£81**  **(-£149 to £310)** |
| ***Scenario 6 (sensitivity 0.55 and specificity 0.92)*** | | | | | |
| Current practice | £16,697  (£327) | --- | 12.00139  (0.00311) | --- | £283,338  (£337) |
| Attentive CTG | £16,611  (£328) | -£86  (-£154 to -£17) | 12.00188  (0.00308) | 0.00049  (-0.00791 to 0.00889) | £283,439  (£338) |
| ***Incremental net monetary benefit (95% confidence interval)*** | | | | | **£98**  **(-£133 to £329)** |
| ***Scenario 7 (sensitivity 0.57 and specificity 0.92)*** | | | | | |
| Current practice | £16,697  (£327) | --- | 12.00139  (0.00311) | --- | £283,338  (£337) |
| Attentive CTG | £16,661  (£328) | -£85  (-£154 to -£17) | 12.00193  (0.00307) | 0.00054  (-0.00784 to 0.00892) | £283,437  (£338) |
| ***Incremental net monetary benefit (95% confidence interval)*** | | | | | **£99**  **(-£131 to £330)** |
| ***Scenario 8 (sensitivity 0.60 and specificity 0.92)*** | | | | | |
| Current practice | £16,697  (£327) | --- | 12.00139  (0.00311) | --- | £283,338  (£337) |
| Attentive CTG | £16,612  (£328) | -£85  (-£154 to -£16) | 12.00201  (0.00306) | 0.00062  (-0.00774 to 0.00898) | £283,438  (£338) |
| ***Incremental net monetary benefit (95% confidence interval)*** | | | | | **£100**  **(-£130 to £331)** |

SE standard error; CTG cardiotocography

**Figure S1 Graph showing 10,000 simulated mean cost and QALY differences between attentive CTG and usual practice on the cost-effectiveness plane** *(circles suggest that attentive CTG is cost-effective)*


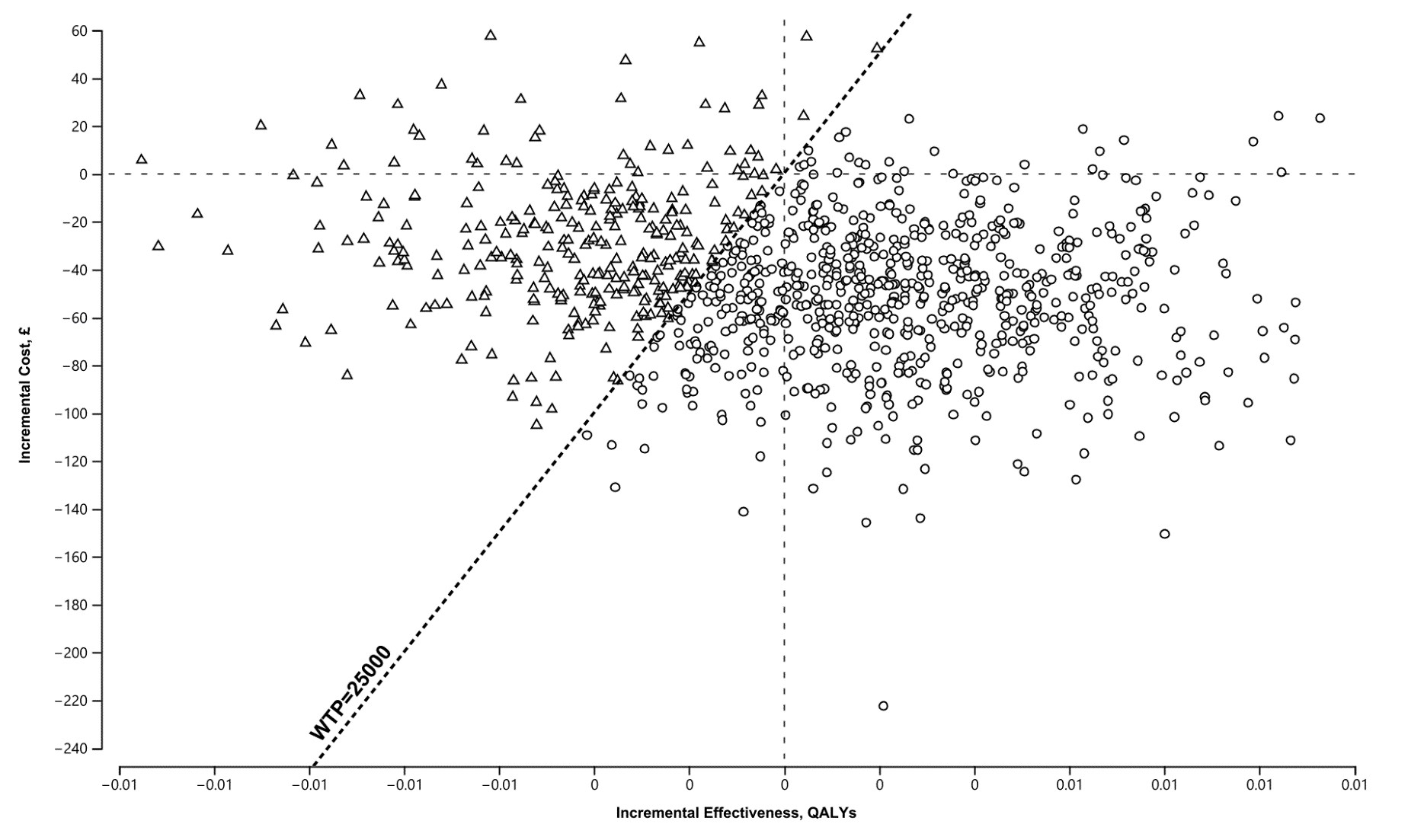


# Reference list

1. Royal College of Obstetricians and Gynaecologists. Each Baby Counts: 2020 Final Progress Report. London: RCOG 2021.

2. Azzopardi D, Brocklehurst P, Edwards D, et al. The TOBY Study. Whole body hypothermia for the treatment of perinatal asphyxial encephalopathy: a randomised controlled trial. BMC Pediatr. 2008;8:17. <https://doi.org/10.1186/1471-2431-8-17>

3. Office for National Statistics. Child mortality (death cohort) tables in England and Wales. 2020. <https://www.ons.gov.uk/peoplepopulationandcommunity/birthsdeathsandmarriages/deaths/datasets/childmortalitystatisticschildhoodinfantandperinatalchildhoodinfantandperinatalmortalityinenglandandwales>. Accessed 01 May 2023

4. Azzopardi D, Strohm B, Marlow N, et al. Effects of hypothermia for perinatal asphyxia on childhood outcomes. N Engl J Med. 2014;371(2):140-9. <https://doi.org/10.1056/NEJMoa1315788>

5. Hemming K, Hutton JL, Bonellie S, Kurinczuk JJ. Intrauterine growth and survival in cerebral palsy. Arch Dis Child Fetal Neonatal Ed. 2008;93(2):F121-6. <https://doi.org/10.1136/adc.2007.121129>

6. Office for National Statistics. National life tables – life expectancy in the UK: 2018 to 2020. 2021. <https://www.ons.gov.uk/peoplepopulationandcommunity/birthsdeathsandmarriages/lifeexpectancies/bulletins/nationallifetablesunitedkingdom/2018to2020>. Accessed 01 May 2023

7. Office for National Statistics. Deaths registered in England and Wales. 2020. <https://www.ons.gov.uk/peoplepopulationandcommunity/birthsdeathsandmarriages/deaths/datasets/deathsregisteredinenglandandwalesseriesdrreferencetables>. Accessed 01 May 2023

8. Petrou S, Kupek E. Estimating preference-based health utilities index mark 3 utility scores for childhood conditions in England and Scotland. Med Decis Making. 2009;29(3):291-303. <https://doi.org/10.1007/s00521-011-0743-y10.1177/0272989x08327398>

9. Pogany L, Barr RD, Shaw A, Speechley KN, Barrera M, Maunsell E. Health status in survivors of cancer in childhood and adolescence. Qual Life Res. 2006;15(1):143-57. <https://doi.org/10.1007/s11136-005-0198-7>

10. Petrou S, Johnson S, Wolke D, Marlow N. The association between neurodevelopmental disability and economic outcomes during mid-childhood. Child Care Health Dev. 2013;39(3):345-57. <https://doi.org/10.1111/j.1365-2214.2012.01368.x>

11. Licchetta M, Stelmach M. Fiscal sustainability analytical paper: Fiscal sustainability and public spending on health. London: Office for Budget Responsibility 2016.

12. NHS England. National Cost Collection National Schedule of NHS costs. NHS trust and NHS foundation trusts. 2020-21. <https://www.england.nhs.uk/publication/2020-21-national-cost-collection-data-publication/>. Accessed 01 May 2023

13. NHS. NHS vaccinations and when to have them. 2023. <https://www.nhs.uk/conditions/vaccinations/nhs-vaccinations-and-when-to-have-them/>. Accessed 01 May 2023

14. NHS. Your baby’s health and development reviews. 2020. <https://www.nhs.uk/conditions/baby/babys-development/height-weight-and-reviews/baby-reviews/>. Accessed 01 May 2023

15. Cecil E, Bottle A, Ma R, et al. Impact of preventive primary care on children's unplanned hospital admissions: a population-based birth cohort study of UK children 2000-2013. BMC Med. 2018;16(1):151. <https://doi.org/10.1186/s12916-018-1142-3>

16. Jones KC, Burns A. Unit Costs of Health and Social Care 2021. Unit Costs of Health and Social Care. Kent: Personal Social Services Research Unit 2021.

17. Regier DA, Petrou S, Henderson J, et al. Cost-effectiveness of therapeutic hypothermia to treat neonatal encephalopathy. Value Health. 2010;13(6):695-702. <https://doi.org/10.1111/j.1524-4733.2010.00731.x>

18. Campbell HE, Kurinczuk JJ, Heazell A, Leal J, Rivero-Arias O. Healthcare and wider societal implications of stillbirth: a population-based cost-of-illness study. BJOG : An International Journal of Obstetrics & Gynaecology. 2018;125(2):108-17. <https://doi.org/10.1111/1471-0528.14972>

19. Gale C, Statnikov Y, Jawad S, Uthaya SN, Modi N. Neonatal brain injuries in England: population-based incidence derived from routinely recorded clinical data held in the National Neonatal Research Database. Arch Dis Child Fetal Neonatal Ed. 2018;103(4):F301-f6. <https://doi.org/10.1136/archdischild-2017-313707>

20. Magro M, Fellow D. Five years of cerebral palsy claims A thematic review of NHS Resolution data. 2017. <https://resolution.nhs.uk/wp-content/uploads/2017/09/Five-years-of-cerebral-palsy-claims_A-thematic-review-of-NHS-Resolution-data.pdf>. Accessed 01 September 2023

21. Office for National Statistics. Birth characteristics in England and Wales 2021. <https://www.ons.gov.uk/peoplepopulationandcommunity/birthsdeathsandmarriages/livebirths/datasets/birthcharacteristicsinenglandandwales>. Accessed 01 May 2023

22. NHS Digital. NHS Maternity Statistics, England - 2020-21. 2021. <https://digital.nhs.uk/data-and-information/publications/statistical/nhs-maternity-statistics/2020-21>. Accessed 01 May 2023

23. Elizabeth S Draper IDG, Lucy K Smith, Alan C Fenton, Jennifer J Kurinczuk, Peter W Smith, Thomas Boby, Bradley N Manktelow on behalf of the MBRRACE-UK collaboration. MBRRACE-UK Perinatal Mortality Surveillance Report UK Perinatal Deaths for Births from January to December 2019. Leicester: The Infant Mortality and Morbidity Studies, Department of Health Sciences, University of Leicester 2021.
